# G Protein-Coupled Receptors: A Century of Research and Discovery

**DOI:** 10.1161/CIRCRESAHA.124.323067

**Published:** 2024-06-20

**Authors:** Samuel Liu, Preston J. Anderson, Sudarshan Rajagopal, Robert J. Lefkowitz, Howard A. Rockman

**Affiliations:** 1 Department of Medicine, Duke University Medical Center; 2 Cell and Molecular Biology (CMB), Duke University, Durham, NC, 27710, USA; 3 Duke Medical Scientist Training Program, Duke University, Durham, NC, 27710, USA; 4 Deparment of Biochemistry Duke University, Durham, NC, 27710, USA; 5 Howard Hughes Medical Institute, Duke University Medical Center, Durham, North Carolina 27710, USA.

**Keywords:** G protein-coupled receptors, biased signaling, allosteric modulators, Basic Science Research, Cell Signaling/Signal Transduction, heart failure

## Abstract

G protein-coupled receptors (GPCRs), also known as 7 transmembrane domain receptors, are the largest receptor family in the human genome, with approximately 800 members. GPCRs regulate nearly every aspect of human physiology and disease, thus serving as important drug targets in cardiovascular disease. Sharing a conserved structure comprised of seven transmembrane α-helices, GPCRs couple to heterotrimeric G-proteins, GPCR kinases and β-arrestins, promoting downstream signaling through second messengers and other intracellular signaling pathways. GPCR drug development has led to important cardiovascular therapies, such as antagonists of β-adrenergic and angiotensin II receptors for heart failure and hypertension, and agonists of the glucagon-like peptide-1 receptor for reducing adverse cardiovascular events and other emerging indications. There continues to be a major interest in GPCR drug development in cardiovascular and cardiometabolic disease, driven by advances in GPCR mechanistic studies and structure-based drug design. This review recounts the rich history of GPCR research, including the current state of clinically used GPCR drugs, and highlights newly discovered aspects of GPCR biology and promising directions for future investigation. As additional mechanisms for regulating GPCR signaling are uncovered, new strategies for targeting these ubiquitous receptors hold tremendous promise for the field of cardiovascular medicine.

## Early history of receptor biology

**Figure 1:**
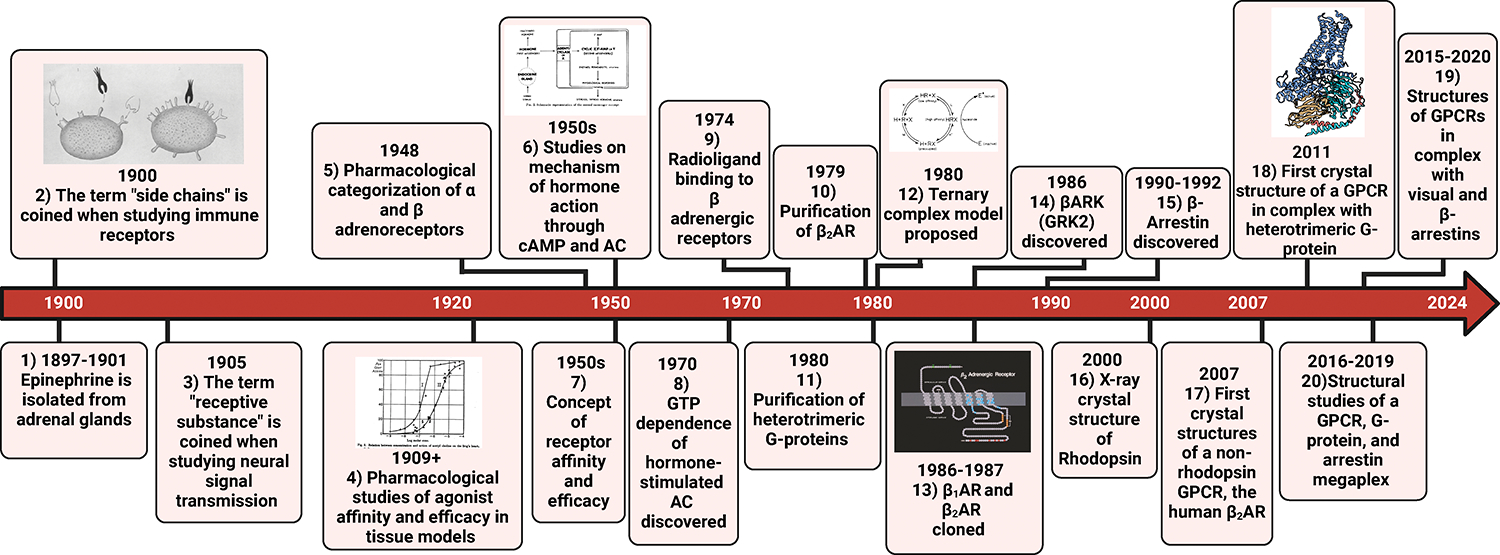
Notable Ligand, Receptor, and Transducer Discoveries. Over the past century, there have been many discoveries from ligands to transducers. Here we have highlighted key studies that have contributed to our knowledge of GPCRs. Below are the corresponding publications for each discovery. Images were reproduced with permission from reference #’s **1.** 1897–1901: Epinephrine is characterized and isolated from adrenal glands.^4,241^ **2.** 1900: The term “side chains” is coined when studying immune receptors.^7^ **3.** 1905: The term “receptive substance” is coined when studying neural signal transmission.^9^ **4.** 1909+: Pharmacological studies of agonist affinity and efficacy in tissue models.^15–18^ **5.** 1948: Pharmacological categorization of α and β adrenoreceptors.^242^ **6.** 1950s: Studies on the mechanism of hormone action through cAMP and Adenylyl Cyclase (AC). ^21,23,243^ **7.** 1950s: Concept of receptor affinity and efficacy.^19,20^ **8.** 1971: GTP dependence of hormone-stimulated AC discovered.^24–26^ **9.** 1974: Radioligand binding of β adrenergic receptors.^31–33^ **10.** 1979: Purification of the β_2_AR.^36^ **11.** 1980: Purification of heterotrimeric G-proteins.^27,28^ **12.**1980: Ternary complex proposed.^35^ **13.** 1986–8: β_1_AR and β_2_AR cloned.^37,244,245^ **14.** 1986: βARK (GRK2) discovered.^38^ **15.** 1990–1992: β-arrestins discovered.^39,40^ **16.** 2000: X-ray crystal structure of Rhodopsin.^246^ **17.** 2007: First crystal structures of a non-rhodopsin GPCR, the human β_2_AR.^191,192^ **18.** 2011: First crystal structure of a GPCR in complex with heterotrimeric G-protein.^193^ **19.** 2015–2020: Structures of GPCRs in complex with visual and β-arrestins.^247–249^ **20.** 2016–2019: Structural studies of a GPCR, G-protein, and arrestin megaplex. ^250,251^

**Figure 2:**
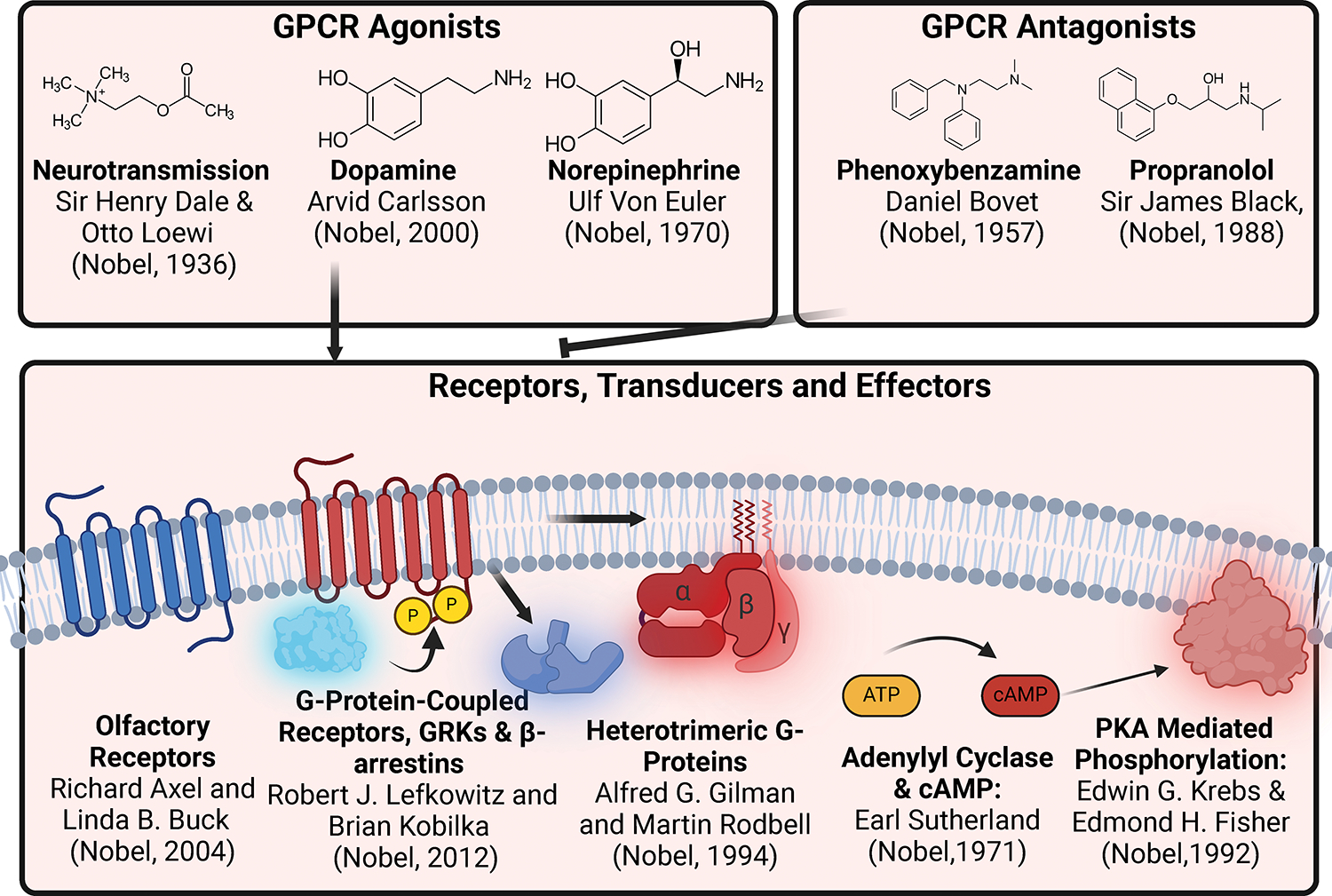
History of Nobel Prize winning GPCR Discoveries The discoveries of components of the GPCR signaling system, ranging from ligands, receptors, and transducers have resulted in numerous Nobel Prizes being awarded. These include the development of clinically important agonists and antagonists, and basic discoveries related to the signaling mechanisms of GPCRs and their transducers. Names and Nobel Prizes were collected from the Nobel Foundation website (https://www.nobelprize.org/prizes/lists/all-nobel-prizes/)

The tantalizing idea that diseases could be treated with specific chemical substances dates to ancient times and traditions across multiple continents and cultures, but for much of history, the exact mechanism by which these substances acted remained a mystery. In the present day, we now know that one large family of over 800 receptors and their transducers, G protein-coupled receptors (GPCRs), make up the targets of ~30% of all FDA-approved drugs.^1,2^ The discoveries surrounding the family of GPCRs are the culmination of pioneering work from countless scientists throughout the 20^th^ century into the modern day, which have resulted in multiple, paradigm-defining discoveries, and Nobel Prizes and which continue to serve as the foundations for innovative fields of biology research today (Figure 1 and 2).

Among the hundreds of clinically targeted GPCRs, the discoveries surrounding the adrenoreceptors, receptors fundamental to normal cardiovascular physiology, make them an excellent case study. The use of naturally existing adrenergic receptor ligands, such as the alkaloid ephedrine from the herb *Ephedra*, can be traced back to ancient Asia.^3^ Epinephrine itself, one of the first human hormones to be isolated, was first isolated as a pure crystal from adrenal glands in 1901^4^ and initially marketed as a wonder drug.^5,6^ Though such naturally occurring adrenergic ligands would see widespread use for centuries, an understanding of their mechanism of action, and of the actual adrenergic receptors they acted on, would only come much later.

Some of the first inquiries in the study of receptors were made by two scientists at the start of the 1900s, Paul Ehrlich and John Langley. Over the course of studying immune responses to pathogens and toxins, Ehrlich would develop a theory of “side-chains,” structures on the surface of cells capable of binding to certain toxins.^7,8^ Just a few years later the earliest assertion of receptor function was made by John. N. Langley, who coined the term “receptive substance” in 1905 while studying the contractions of muscle cells stimulated by nicotine. He describes:

“So we may suppose that in all cells two constituents at least are to be distinguished. The chief substance which is concerned with the chief function of the cell as contraction and secretion and receptive substances which are acted upon by chemical bodies and in certain cases by nervous stimuli. The receptive substance affects or is capable of affecting the metabolism of the chief substance”^9^

Though the identities of the “receptive substances” were a mystery to Langley, these early ideas led to the initial proposal of the receptor concept. The functions of receptors, Langley had clearly postulated, are: first, to interact with external chemical stimuli; and second, to relay these responses to effectors within the cell, generating physiological changes in response. Despite the later-proven accuracy of this theory, these ideas were initially met with considerable skepticism, and the existence of receptors would remain highly contentious for decades. For instance, consider the perspectives of just a few scientists in the following decades.

In 1943, Sir Henry Dale, who received the Nobel Prize in 1936 for studies on adrenergic and cholinergic neurotransmission and himself a former student of John Langley would say:

“It is a mere statement of fact to say that the action of adrenaline picks out certain such effector cells and leaves others unaffected; it is a simple deduction that the affected cells have a special affinity of some kind for adrenaline, but I doubt whether the attribution to such cells of “adrenaline receptors” does more than restate this deduction in another form….”^10^

In 1973, Raymond Ahlquist, a distinguished pharmacologist, and recipient of the Lasker prize for his work on the pharmacological differentiation of *α* and *β* adrenoreceptor subtypes would remark:

“This would be true if I were so presumptuous as to believe that α and β receptors really did exist. There are those that think so and even propose to describe their intimate structure. To me they are an abstract concept conceived to explain observed responses of tissues produced by chemicals of various structure.”^11^

The 1955 edition of The Pharmacological Basis of Therapeutics, a standard textbook of pharmacology, states: *“Years ago, Langley named the differentiating substance the ‘receptive substance’; this term is still widely employed, but it must be realized that the ‘receptor’ may not be a morphologically demonstrable structure.”*^12^ Taken together, these quotes illustrate the dogma in the field at the time.

Despite these limitations to the basic understanding of receptors, an understanding of the physicochemical basis of ligand binding to receptors began to emerge after Langley’s initial work, leading to the development of “receptor theory.”^13,14^ Through the work of Hill (who worked with Langley),^15^ Clark,^16^ and others, physicochemical models for understanding ligand binding to receptors emerged by developing quantitative relationships between drug action and changes in physiology. Work through the 1950s led by Gaddum,^17^ Schild,^18^ Ariens,^19^ and Stephenson^20^ led to the important concepts of affinity, reflecting how tightly a drug bound to its receptor, and efficacy, how effectively the drug:receptor complex promoted a physiological response. But despite these advances, the molecular mechanisms underlying this drug action largely remained a black box.^13^

Insights into the mechanisms of drug action started to change with the discovery of the intracellular signaling pathways regulated by receptors. Research from Earl Sutherland Jr.’s lab first showed that the activity of β-adrenoreceptors resulted in the production of a “second messenger”, cyclic adenosine monophosphate (cAMP), which was later shown to be produced by adenylyl cyclase (AC).^21–23^ Sutherland’s work on AC would be further built upon by the work of Martin Rodbell’s lab which determined that AC activation by hormones required the presence of guanosine triphosphate (GTP), presumably through the association of an intermediary protein that bound GTP.^24–26^ These discoveries would be further built on by Alfred Gilman’s lab, which successfully identified and isolated the intermediary heterotrimeric G-protein.^27,28^ For this work, Sutherland received the Nobel Prize in 1971 and Rodbell and Gilman shared the Nobel Prize in 1994.

## β-adrenergic receptors as the prototype for GPCRs

Among the many GPCRs, β-adrenergic receptors were used as the model system in many early studies to understand GPCR structure and function. The importance of β-adrenergic signaling in cardiovascular physiology led to the development of antagonists of the β-adrenergic receptor, commonly referred to as “beta-blockers.” With the hypothesis that inhibiting adrenergic signaling would diminish the heart’s demand for oxygen in the setting of angina, Sir James Black led the development of the first beta-blocker in the 1960s, at a time without knowledge of receptor structure or how adrenergic ligands bound to the receptor, and when the existence of receptors themselves was still questionable. Black ultimately identified the first clinically utilized beta-blocker, propranolol, synthesizing and screening numerous derivatives of catecholamines, applying mathematical models of affinity and efficacy to identify competitive antagonists for the receptor.^29,30^

The development of the first small-molecule antagonists paved the way for the use of ligands as tools to label, capture, and purify adrenergic receptors themselves, work which was pioneered in the Lefkowitz laboratory. Radiolabeling β-adrenergic-specific agonists and antagonists provided a highly specific method for detecting receptors.^31–33^ The binding of agonists to receptors was found to be biphasic, showing two distinct affinity states (high and low) due to the allosteric effects of G-proteins on the receptors. Notably, the addition of guanine nucleotides converted high to low-affinity state receptors (by promoting heterotrimeric G-protein dissociation).^34^ This ultimately led to the development of the ternary complex model to explain the allostery between receptors, agonists, and heterotrimeric G-proteins.^35^ Concurrently, work on chemical conjugation of the beta-blocker, alprenolol, onto gel columns allowed for the development of affinity chromatography techniques to capture and purify the β2-adrenergic receptor (β_2_AR), one of the three β-adrenergic receptors expressed in the heart.^36^ This in turn ultimately facilitated the cloning of the β_2_AR gene.^37^ Purified receptors were chemically cleaved into short peptides, several of whose amino acid sequences were determined, thus permitting the design of oligonucleotide probes which were used to clone the receptor gene and cDNA.^37^ The cloning of the β_2_AR was a watershed event, since it revealed the similarity of its primary structure and seven transmembrane domain architecture to the retinal “light receptor” rhodopsin. In the years after, several additional GPCRs, including the β_1_AR, the predominant β-adrenergic receptor expressed in the heart, would also be cloned using a similar strategy. Subsequently, hundreds of receptors would be shown to share these features and these insights marked the discovery of the superfamily of seven transmembrane receptors that regulate virtually all of human physiology.

Rapid progress in understanding the mechanisms regulating GPCR signaling was made in the Lefkowitz lab during the 1980s and 1990s. After heterotrimeric G-proteins are activated by GPCRs, the receptors rapidly “desensitize,” preventing uncontrolled signaling acutely, and then in the face of prolonged signaling “downregulate,” due to their destruction in lysosomes with decreased receptor expression to decrease further signaling and maintain homeostasis. This process was shown to require receptor phosphorylation and through analogy with rhodopsin, kinases that we now refer to as GPCR kinases (GRKs).^38^ Desensitization further requires the activity of adapter proteins known as β-arrestin-1^39^ and 2^40^, which share homology with a retinal protein originally known as S-antigen (now known as visual arrestin) which had been shown to quench rhodopsin signaling.^41^ This family of 4 proteins consists of arrestins 1 and 4 with expression restricted to the retina, and β-arrestin-1^39^ and 2^40^ (aka arrestins 3 and 4) which are expressed ubiquitously. β-arrestins were also shown to promote endocytosis and downregulation of most receptors.^42,43^ Despite their initial discovery of “arresters” of signaling, β-arrestins were later shown to also promote signaling through kinase cascades^44^ and other mechanisms, displaying their multifunctional nature in the regulation of receptor desensitization, trafficking, and signaling.

Of the 3 βARs subtypes (β1AR, β2AR, and β3AR) expressed in the heart, β1ARs are the most abundant (~75–80%) with some expression of β2ARs (~15–20%) and minimal expression of β3AR.^45,46^ Subsequent discoveries led to additional understanding of their roles in the pathophysiology of heart disease, such as the observations of beta-receptor downregulation^45,47^ and altered expression of GRKs^48^ in heart failure. Studies investigating levels of beta receptor ligands found them useful as biomarkers for HF progression, ultimately culminating in the landmark paper in 1984 proposing the neurohormonal hypothesis of HF: the idea that adverse cardiac remodeling and progression of heart failure is dependent on overactivation of the autonomic sympathetic nervous system.^49^ Subsequent work in experimental systems found that transgenic mice with elevated β_1_AR signaling, either through β_1_AR overexpression^50^ or Gα_s_ overexpression,^51^ spontaneously develop heart failure while transgenic mice that overexpress β_2_AR in the heart display enhanced cardiac function.^52^

The increasing understanding of βAR dysfunction in heart failure came at a time when there was a long-standing belief that beta-blockers were contraindicated in the setting of heart failure due to their negative inotropic properties. Over time, these views became increasingly challenged, and several major trials in the 1980s and 1990s conclusively demonstrated that beta-blockers substantially improved survival,^53–57^ although this effect was only observed in beta-blockers that did not have weak partial agonist activity, known as “intrinsic sympathomimetic activity”.^58–60^ Beta-blockers would prove to be immensely successful in the treatment of heart failure, and the story of these adrenoreceptor ligands illustrates the rich history of this family of receptors, the dramatic advances in their understanding over the past century, and the immense therapeutic potential that they hold.

## Molecular machinery underlying GPCR signaling

**Figure 3:**
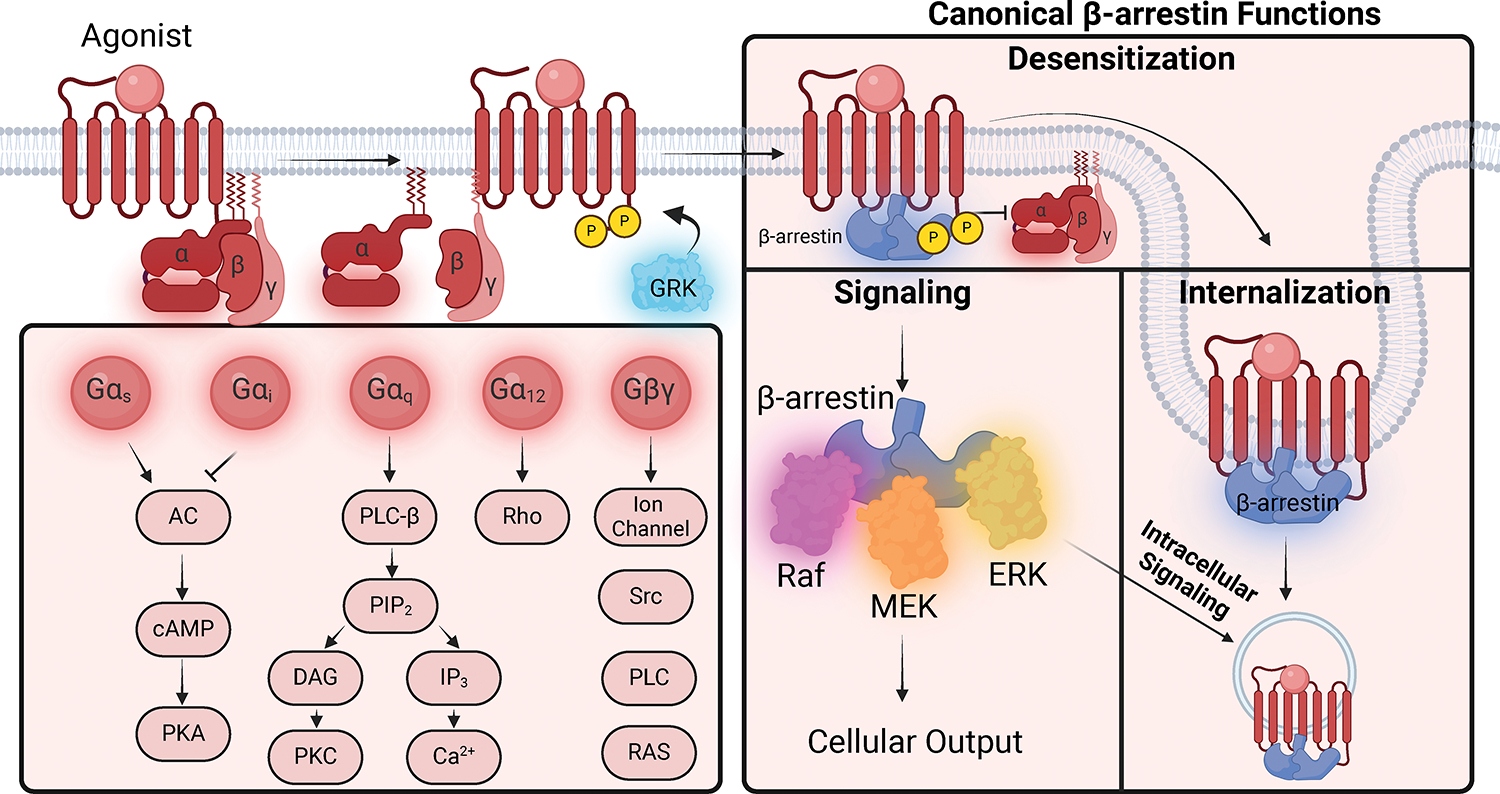
GPCR signaling via G proteins, GRKs and β-arrestins. Upon agonist stimulation, heterotrimeric G-proteins (Gα, Gβ, and G𝛾) are recruited to the receptor and there is guanine nucleotide exchange of GTP for GDP. The Gα-GTP subunit dissociates from the Gβ𝛾 transducer, and both signal to diverse downstream effectors. Gα has four distinctive families (Gα_s_, Gα_i,_ Gα_q,_ Gα_12/13_). GRKs phosphorylate the intracellular domains of the GPCR, which promote tight binding of β-arrestins. β-arrestins have three canonical functions: receptor desensitization, internalization, and signaling. In addition, there is intracellular signaling of β-arrestins from endosomes. GRK, G-protein receptor kinase; GDP, Guanosine diphosphate; GTP, Guanosine triphosphate; AC, adenyl cyclase; ATP, adenosine triphosphate; cAMP, cyclic adenosine monophosphate; PKA; Protein kinase A; PLC- β, phospholipase C- β; IP_3_, inositol trisphosphate; PIP_2_ phosphatidylinositol 4,5-bisphosphate; DAG, diacylglycerol; PKC, Protein kinase C; ERK, extracellular signaling-related kinases

GPCRs share a conserved 7 transmembrane domain structure with an extracellular-facing ligand binding site and an intracellular pocket for transducer binding. GPCRs also have 3 intracellular loops and a C-terminal tail which regulate transducer interactions. GPCRs are activated by ligand binding to the GPCR, inducing conformational changes that allow for the subsequent recruitment of effectors. GPCRs signal through a variety of effectors, most prominently heterotrimeric G-proteins, GRKs, and β-arrestins (Figure 3).

### Heterotrimeric G proteins

Heterotrimeric G-proteins, the largest family of GPCR signaling transducers, are composed of a complex of G⍺, G_β_, and G𝛾 subunits. Upon receptor activation, the receptor acts as a guanine nucleotide exchange factor for the G⍺ subunit, inducing release of guanosine diphosphate (GDP) in exchange for guanosine triphosphate (GTP). This induces dissociation of the G⍺-GTP subunit from the G_β𝛾_ complex, allowing each of these units to independently interact with a wide range of effectors to regulate second messenger levels, protein kinases, and other pathways to impact different cellular functions.^61^

There are sixteen Gα subunits that fall into four main families: Gα_s,_ G⍺_i_, G⍺_q/11,_ G⍺_12/13,_ all of which differentially engage with a variety of effectors. Different receptors preferentially activate specific Gα subunits allowing for highly specific signaling pathways to occur downstream of receptor activation. G⍺_s_ subunits stimulate AC to convert adenosine triphosphate (ATP) to cAMP and lead to the activation of protein kinase A (PKA)^62^ and other targets. In the heart, PKA phosphorylates a wide array of effectors such as troponin I, myosin-binding protein-C, phospholamban, the cardiac ryanodine receptor (RYR2), and voltage-gated L-type Ca^2+^ channels.^63^ βAR stimulation collectively orchestrates these subcellular components to enhance myofilament cross-bridge cycling to increase the force of contraction (inotropy) and the rate of relaxation (lusitropy). Action potential activated L-type Ca^2+^ channels initiate inward Ca^2+^ entry and trigger a large release of intracellular Ca^2+^ by the RYR2 through a process known as Ca^2+^-induced Ca^2+^ release. This triggered Ca^2+^ release by the RYR2 was defined as a Ca^2+^ spark^64^ and is the primary source of intracellular Ca^2+^ available for myofilament cross-bridge formation and contraction. βAR stimulation also regulates heart rate (chronotropy) via the Hyperpolarization-activated Cyclic Nucleotide-gated channel and other ion channels and transporters in the sinoatrial node.^65^ In contrast, receptors that couple through G⍺_i_ inhibit AC, therefore reducing cAMP production and its downstream effects on the heart.

G⍺_q/11_ activates phospholipase-Cβ to convert membrane-bound phosphatidylinositol 4,5-bisphosphate (PIP_2_) to diacylglycerol (DAG) and inositol 1,4,5-trisphosphate (IP_3_). IP_3_ activates cardiomyocyte IP_3_ receptors, which are intracellular Ca^2+^ release channels embedded in the sarcoplasmic reticulum (SR) and nuclear envelope. GPCR stimulated IP_3_ production elicits local nuclear envelope Ca^2+^ release via IP_3_ receptors to activate cardiac hypertrophic signaling, in part, through the Ca2+ and calmodulin-dependent serine/threonine protein phosphatase calcineurin^66^ and a nuclear pool of Ca^2+^-calmodulin–dependent protein kinase II.^67^ IP_3_ stimulated release of this local Ca^2+^ pool is distinct from the cytoplasmic Ca^2+^ transient involved in excitation-contraction coupling^66,67^. DAG and intracellular Ca^2+^ activate protein kinase C-alpha (PKC-a) which regulates cardiac contractility through upregulation of type 1 protein phosphatase leading to dephosphorylation of the SR Ca^2+^ATPase pump regulating protein phospholamban.^68^

G⍺_12/13_ are known to activate the small GTPase RhoA,^69,70^ which can activate downstream kinases, regulating vascular smooth muscle tone.^71–73^ Beyond these canonical signaling pathways, each of these G⍺ subunit families can regulate a wide array of relatively poorly characterized signaling pathways. Furthermore, in addition to the G⍺ subunits, there are 5 Gβ subunits and 12 Gγ subunits, which also regulate a wide range of signaling pathways with distinct spatial and temporal profiles.^74,75^

Heterotrimeric G protein signaling is essential for normal cardiac function but chronic sustained stimulation, particularly of G⍺_s_ and G⍺_q/11_, can lead to deleterious adverse ventricular remodeling and depressed cardiac function. In the setting of the heart, the negative consequences of overactive G-protein signaling include tachycardia and progression of heart failure.^76^ In contrast, activation of G⍺_i_ can play an inhibitory role on AC activity and may be beneficial for opposing G⍺_s_ signaling. Thus, negative feedback loops are crucial in maintaining cellular homeostasis after GPCR stimulation. Active G⍺-GTP is turned off by regulators of G protein signaling (RGS) proteins, which act as GTPase activating proteins (GAPs) that promote hydrolysis of GTP to G⍺-GDP.^77^ This, in turn, leads to the termination of G⍺-protein-mediated signaling and reassociation of the heterotrimeric G-protein complex. Complex mechanisms promote the process of receptor desensitization, in which the ability of active receptors to signal is diminished, otherwise signaling would proceed unabated.

### GRKs and Other Kinases

Desensitization is promoted through receptor phosphorylation by kinases that phosphorylate serine/ threonine motifs in the receptor’s intracellular loops and C-tail. These phosphorylation patterns either directly interfere with the receptor’s normal signaling functions or lead to the recruitment of β-arrestins that sterically interfere with G-protein coupling and induce receptor internalization.

There are two types of processes by which kinases regulate receptor desensitization: homologous desensitization, in which kinases are recruited to and phosphorylate an agonist-activated receptor, or heterologous desensitization, in which kinases phosphorylate specific motifs on receptors regardless of their activation state. Homologous desensitization is promoted primarily by GRKs, which are recruited through an interaction with the core of the active receptor, and which phosphorylate the receptor intracellular loops and C-terminal tail.^78,79^ Heterologous desensitization is mediated primarily by second messenger-dependent kinases such as PKA and PKC, which recognize specific motifs in receptor intracellular domains. Thus, even in the absence of its cognate ligand, a receptor can undergo heterologous desensitization. For example, cAMP or cAMP analogs promote phosphorylation of the β_2_AR through the activity of PKA, thereby reducing the receptor’s ability to signal through G⍺_s_^80^. A related phenomenon is class switching, where phosphorylation changes the coupling specificity of a receptor. Examples include the β_2_AR, where PKA phosphorylation leads to the receptor changing its preferential coupling from G⍺_s_ to G⍺_i_,^81^ further inhibiting signaling through AC. Similarly, for the glucagon-like peptide-1 receptor (GLP-1R), PKC modulates a signaling switch from G⍺_s_ to G⍺_q_.^82^

Regulation of receptor phosphorylation is highly dependent on cellular context. There are 7 GRKs in humans, all of which share a common tripartite structure: a GPCR binding domain, a kinase domain, and a regulatory domain.^83^ Based on structural and sequence homology, they are further subdivided into three subfamilies: the GRK1 family(GRK1 and 7); the GRK2 family (GRK2 and 3); and the GRK4 family (GRK4, 5, and 6). GRK1 and 7 are primarily found in retinal rod and cone cells respectively.^84^ GRK2 and 3 both contain a C-terminal pleckstrin homology (PH) domain, which promotes their localization to the plasma membrane via Gβγ binding.^85^ GRKs 4 and 6 carry palmitoylation sites, and GRK 5 contains a positively charged lipid binding element. As a result, GRK 4, 5, and 6 do not require interaction with G_β_𝛾 to localize to the plasma membrane. Adding to the context-dependent activity of these kinases, different GRKs and PKA/PKC also induce different phosphorylation patterns at the receptor, with distinct impacts on receptor signaling and recruitment of other proteins to the receptor.^86–88^ Taken together, the function of these protein kinases is to fine-tune GPCR signaling responses.

In addition to the GPCRs a number of nonreceptor substrates have been shown to be phosphorylated by or interact with these kinases such as receptor tyrosine kinases, cytoskeletal proteins,^89^ and phosphoinositide 3-kinase (PI3K).^90,91^ PKA and GRK5 can also serve as adapter proteins and translocate to the nucleus,^92–95^ where they can regulate gene expression.

### β-arrestins: Canonical GPCR transducers

Arrestins are a family of four multifunctional adaptor proteins with three main functions at the receptor: desensitization, trafficking, and signaling. There are four arrestins that share high sequence and structural homology, containing an N-terminal domain, a central core domain, and a C-terminal tail. Arrestin 1 (aka visual arrestin) and 4 (aka x-arrestin) are found exclusively in rod and cone cells while β-arrestins 1 and 2 (aka arrestins 2 and 3) are found ubiquitously.^96^ While β-arrestins 1 and 2 share many structural and functional similarities, they also have their own distinct cellular functions. For example, β-arrestin 1 and −2 both have nuclear localization sequences on the N-terminus, but β-arrestin 2 has a nuclear export sequence on the C-terminus resulting in differential nucleocytoplasmic shuttling of the β-arrestins.^97^

Upon receptor phosphorylation, β-arrestins are recruited to the receptor through an interaction with the receptor’s phosphorylated tail. Additional interactions with the intracellular loops and core of activated receptors permit a range of conformational states of β-arrestin:receptor complexes.^98,99^ This recruitment of β-arrestins sterically interferes with receptor interactions with the Gα subunit, desensitizing the receptor by restricting further signaling by G-proteins.

Simultaneously, binding of β-arrestin to a receptor induces conformational changes in β-arrestin, which allow it to interact with hundreds of proteins involved in a wide range of functions.^100,101^ β-arrestin then promotes the process of receptor internalization, decreasing receptor expression at the plasma membrane by serving as an adaptor for the endocytotic machinery through AP2 and clathrin binding motifs found on its C-tail.^43,102,103^ This promotes the translocation of the receptor to intracellular compartments for recycling or degradation.^79^ Receptor:β-arrestin interactions play an important role in specifying the specific targeting of receptors to intracellular compartments. For example, receptors that have high-affinity for β-arrestin, such as the vasopressin 2 receptor (V_2_R) and angiotensin II receptor type 1 (AT_1_R), experience sustained internalization, often proceeding to lysosomes for degradation. In contrast, receptors such as the β_2_AR, which have a relatively low affinity for β-arrestin, are recycled rapidly back to the cell surface after internalization.^102^

Receptor-activated β-arrestin also serves as a scaffold and allosteric activator for protein kinase signaling cascades, amplifying the activity of these pathways within cells. These include mitogen-activated protein kinases (MAPK), PI3K, AKT, and Src^44,90,104–107^ in addition to a wide range of other pathways.^108^ G-protein and β-arrestin-mediated signaling display different spatial and temporal profiles,^109^ although this can vary significantly among receptors. Even when activating the same distal effector, they often mediate different cellular sequelae due to differential subcellular compartmentalization of the activated effectors. For example, the AT_1_R, an important receptor with key roles in blood pressure regulation, vascular function, and cell remodeling signals to ERK through both G protein and β-arrestin transducers. In response to stimulation by the endogenous ligand, angiotensin II (AngII), AT_1_R G⍺_q_-activated ERK translocates to the nucleus, where it activates transcriptional networks. In contrast, β-arrestin sequesters ERK in the cytosol resulting in the phosphorylation of cytosolic proteins resulting in activation of different cellular responses.^109^ In the heart, signaling via β_1_AR-mediated Epidermal Growth Factor Receptor (EGFR) transactivation is cardioprotective against chronic catecholamine stimulation^110^ through a β-arrestin dependent mechanism mediated, in part, through differential intracellular trafficking of ERK1/2.^111^ At the molecular level, there appears to be functional specialization between β-arrestin isoforms. At the AT_1_R, recruitment of β-arrestin 1 and 2 are similar, but siRNA knockdown of β-arrestin 1 results in increased ERK signaling, whereas knockdown of β-arrestin 2 results in decreased ERK signaling. This suggests that β-arrestin 2, but not β-arrestin 1, mediates ERK1/2 activation at the AT_1_R.^112,113^ On the other hand, at the β_2_AR, knockdown of either β-arrestin 1 or 2 results in diminished ERK signaling, suggesting that β-arrestins mediate different responses at different receptors.^106^ While β-arrestin-mediated responses are thought to be distinct from those mediated by G-protein, more recently, it has also been appreciated that β-arrestins can promote G-protein signaling from internalized GPCRs.^114,115^ While β-arrestins were originally identified for their role in desensitizing G protein signaling by GPCRs at the plasma membrane, they can actually promote signaling from internalized receptors. These contrasting functions further highlight the diversity of cellular responses mediated by β-arrestins.

### Other GPCR-binding Proteins

Individual GPCRs are also capable of interacting with a wide range of proteins depending on the presence of specific motifs in their intracellular domains.^116^ These GPCR-interacting partners can sometimes directly mediate receptor signaling or act as scaffolds to modulate signaling. These include the AKAPs, Homer, JAK2, and NHERF1 (reviewed in^116^). One important family of GPCR-interacting proteins is the receptor activity modifying proteins (RAMPs).^117^ The activity of RAMPs greatly modifies the behavior of GPCRs. For the calcitonin-like receptor (CLR), association with RAMP1 results in a receptor for the calcitonin gene-related peptide (CGRP), promoting vasodilation in diseases such as migraine.^118^ This is in contrast to the properties of CLR in association with RAMP2, which generates an adrenomedullin 1 receptor where adrenomedullin has the highest potency for vasodilation, and association with RAMP3, which generates an adrenomedullin 2 receptor where adrenomedullin 2 and adrenomedullin have similar potency.^117^ Originally thought to only interact with a limited number of family B receptors, recent studies have now demonstrated that RAMPs interact with a much wider range of GPCRs.^119,120^ This likely is the tip of the iceberg, as other receptor-interacting proteins likely play additional roles in GPCR biology.

### Biased Agonism

**Figure 4:**
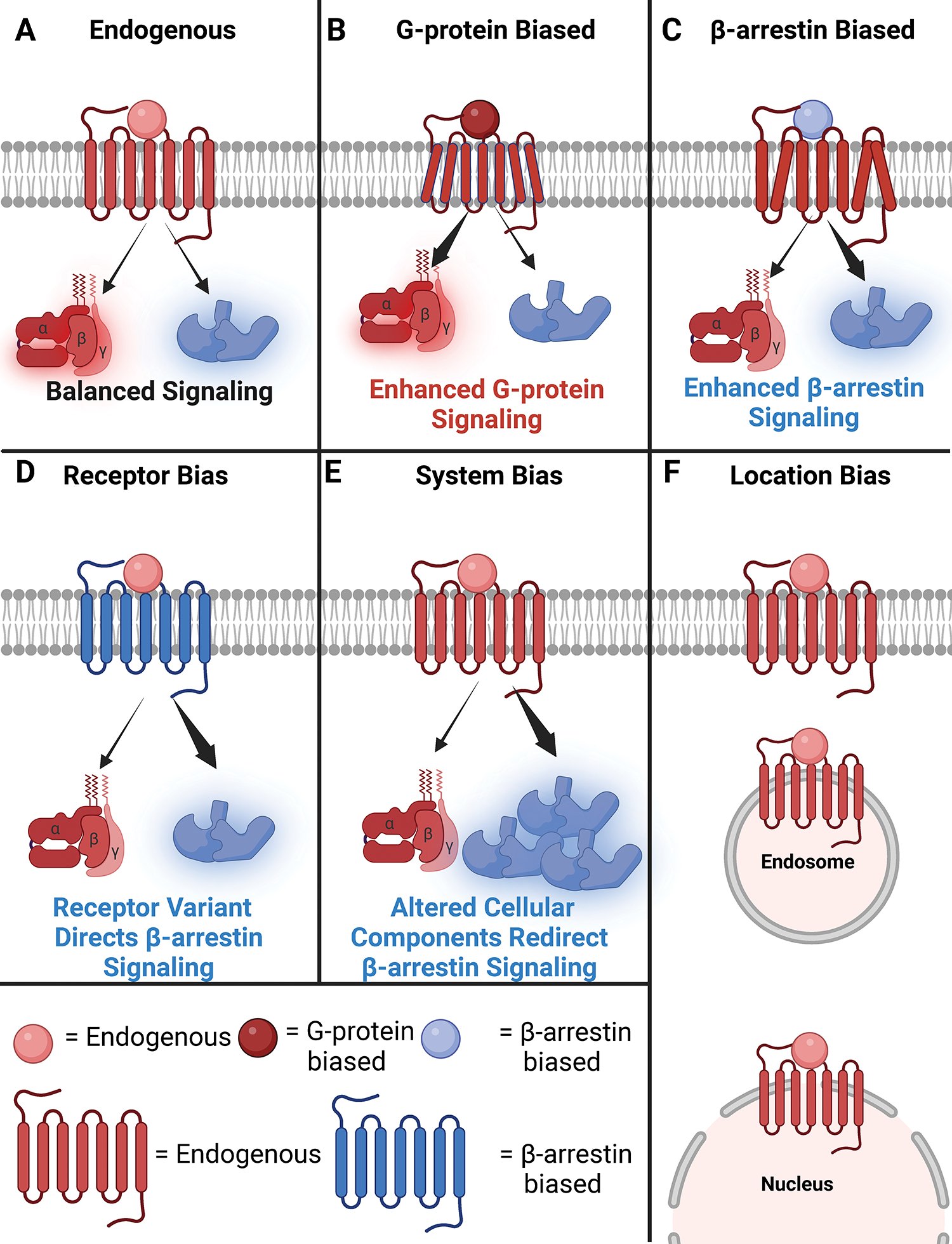
Biased Signaling of GPCRs. (**A**) Reference/endogenous agonists binding to receptors can signal through two different pathways, G-proteins and β-arrestin. (**B**) Ligand bias promotes the receptor:transducer complex to adopt certain conformations that bias the signaling through (**B**) G-proteins or (**C**) β-arrestin. (**D**) Biased receptors may have altered phosphorylation sites on their C-tail or other mutations that may bias signaling toward β-arrestin instead of G-proteins despite being stimulated with an endogenous agonist. (**E**) System bias occurs when there is a differential expression of signaling components. For example, some cells express different isoforms of GRK and β-arrestin and may bias signaling through β-arrestin. (**F**) Location bias refers to receptors promoting signaling from distinct intracellular locations, such as from endosomes, the nucleus, or the plasma membrane.

As GPCRs can signal through multiple pathways promoted by different G proteins, GRKs, β-arrestins, and other interacting proteins, it has been appreciated that in different contexts, the same receptor can promote distinct patterns of signaling outcomes through selectively activating subsets of transducers (Figure 4).^121^ The ability of a GPCR to selectively or preferentially couple to distinct transducers is now referred to as *biased agonism*,^121^ and can be induced through a variety of mechanisms. The first discovered form of bias was *ligand bias,* referring to the phenomenon whereby different ligands for a GPCR were found to have different efficacies for activating downstream transducers, such as preferentially recruiting β-arrestins, leading to a biased response compared to the reference endogenous agonist.^122^ Ligand bias is thought to be due to the ability of different agonists to promote distinct conformational states of the receptor that have different efficacies for engaging with different transducers and initiating signaling through different signaling pathways.^123^ It is now appreciated that all agonists lie on a spectrum of bias through their stabilization of different receptor conformations.^122^

There are additional mechanisms that may underlie a biased cellular response, such as *receptor*, *system,* and *location* bias. *Receptor* bias (Figure 4) refers to bias at the level of the receptor itself, where a receptor inherently couples more effectively to one transducer or another compared to a reference. The existence of such receptors can be demonstrated by experimentally generated receptors where important residues for receptor activation and transducer coupling have been mutated such as the AT_1_R “AAY” or β_2_AR “TYY” mutant receptors which show biased coupling preferences compared to their unmodified wild-type receptors.^106,112^ Additionally, naturally existing receptor variants that lead to biased signaling profiles have also been identified in a number of disease states.^124^ *System bias* (Figure 4), refers to the differential expression of individual signaling components, such as G proteins, β-arrestins, and GRKs, which can lead to tissue/cell type-specific differences in signaling by the same agonist:receptor complex.^121^ Such situations can occur in disease states where there is differential expression of receptors, transducers, and effectors.^125,126^ A related concept is that of *location bias* (Figure 4), where different ligands promote receptor activation in different subcellular locations, resulting in distinct cellular and physiological effects.^127^

As bias allows for the selection of signaling pathways to be activated downstream of a receptor, biased ligands have the potential to activate beneficial signaling pathways while limiting off-target effects at a given GPCR. Thus, drug development of biased agonists is a very active area of research. For example, activation of the AT_1_R by its endogenous ligand AngII in rats initiates signaling through both G-protein and β-arrestin dependent pathways, enhancing cardiac contractility while inducing cardiac hypertrophy. In constrast the synthetic, β-arrestin biased AT_1_R agonist TRV027 enhances contractility but does not induce cardiac hypertrophy.^128^ Bias is also highly pertinent to the elucidation of GPCR function in their endogenous context, particularly location bias and system bias. For example in cardiomyocytes, numerous receptors are expressed, such as βAR, ⍺1AR, and AT_1_R, in various cellular compartments and the nucleus, where this spatial localization contributes to their unique signaling functions.^129–131^ These topics will be further explored in the **Emerging Directions** section at the end of the review.

## Important GPCR Targets in Cardiovascular Medicine

There are more than 200 GPCRs expressed in the heart,^132^ and drugs targeting many of these GPCRs expressed in the cardiovascular system are mainstays of clinical treatment for a wide range of pathologies. Here we highlight three families of GPCRs with historical, clinical, and emerging importance in cardiovascular medicine: βARs, AT_1_Rs, and incretin receptors (Table 1).

### β-Adrenergic Receptors

The β_1_AR and β_2_AR are the predominant adrenergic receptor subtypes expressed in the heart, while the β_3_AR is primarily expressed in adipose tissue. In the normal heart, the β_1_:β_2_ ratio is 80:20,^45^ while in heart failure, the β_1_:β_2_ ratio is reduced to 60:40, due to the downregulation of the β_1_AR.^47^ In cardiac myocytes, β_1_ARs predominate with β_2_ and β_3_ARs mostly found in nonmyocytes.^46^ Upon stimulation with its endogenous ligands epinephrine and norepinephrine, the β_1_AR couples to G⍺_s,_ the β_2_AR to G⍺_s_ and G⍺_i_, and the β_3_AR to G⍺_s_. Activation of βARs generally initiates a Gs-AC-cAMP-PKA signaling cascade, increasing myocardial contraction and heart rate. However, long-term activation of G⍺_s_ signaling by βARs in heart failure can lead to pathological remodeling of heart tissue and their downregulation.^133^

As noted earlier, beta-blockers are one of the most widely used therapeutics for numerous diseases including hypertension, coronary artery disease, heart failure, and arrhythmias.^54,134^ However, initially, the use of beta-blockers for heart failure was seen as a contraindication, and it took nearly 30 years from the discovery of beta-blockers to the first clinical trials for use in heart failure. In the 1990’s, some of the earliest beta-blocker clinical trials of metoprolol were associated with lower mortality rates^135^ (Table 1). This reinvigorated interest in beta-blockers, and a third-generation beta-blocker, carvedilol, was shown to reduce cardiovascular-related hospitalization and death.^54^ Although carvedilol is an FDA-approved beta-blocker, the complexities of its pharmacology are still being uncovered. At the β_2_AR, carvedilol is a β-arrestin-biased agonist with inverse agonism for G⍺_s_ signaling.^136^ At the β_1_AR, carvedilol displays β-arrestin-biased signaling dependent on G⍺_i_, regulates microRNA processing, activates ERK signaling,^137–139^ and provides cardioprotection in response to ischemia-reperfusion.^140^ β_1_AR mediated β-arrestin transactivation of the epithelial growth factor receptor (EGFR), requires β-arrestin, GRK5 and/or 6.^110^ Activation of this transactivation pathway is cardioprotective against catecholamine toxicity.^110^ Though it has been reported that carvedilol can activate G⍺_s_,^141^ such findings have not been observed in most physiologically-relevant systems and run counter to numerous clinical studies showing that beta-blockers with intrinsic sympathomimetic activity are associated with negative outcomes in heart failure.^58–60^ Furthermore, carvedilol also has additional α1 adrenergic blockade and antioxidant properties, which have all been theorized to contribute to its unique cardioprotective properties.^142,143^ At the moment, the question of what specific properties will result in the development of “better” beta-blockers is still under investigation, though research in our laboratories has focused on evaluating and enhancing the effects of β-arrestin-biased signaling of beta-blockers.^140,144^

### The Type 1 Angiotensin II Receptor

The renin-angiotensin-aldosterone system (RAAS) plays a central role in regulating blood pressure and volume status through its effects on the cardiovascular system and kidneys.^145^ Renin cleaves angiotensinogen into angiotensin I, which is further processed by angiotensin-converting enzyme in the lungs into AngII. The angiotensin II receptor type 1 (AT_1_R) and type 2 (AT_2_R) appear to have contrasting physiological roles. In the cardiovascular system, the AT_1_R promotes vasoconstriction, and vascular smooth muscle cell proliferation, while AT_2_Rs promote vasodilatation and inhibit proliferation. In heart failure, AT_1_R mRNA expression is downregulated, whereas there is no change for AT_2_R.^146^ AT_1_R signaling also promotes the secretion of aldosterone by the adrenal cortex and promotes sodium retention in the kidney.

AT_1_Rs can signal through a wide range of pathways.^147–150^ Upon activation by its endogenous ligand, AngII, the AT_1_R signals through G⍺_q_, but can also couple to other G proteins, such as G⍺_12/13_ and G⍺_i/o_. In addition, the AT_1_R can signal via β-arrestins to multiple effectors, such as MAP kinases. Furthermore, the AT_1_R is also capable of promoting signaling through other transmembrane receptors, such as the EGFR through β-arrestin-dependent and -independent mechanisms.^151–153^

FDA-approved RAAS inhibitors include direct renin inhibitors, angiotensin-converting enzyme inhibitors (ACEIs), AT_1_R antagonists (ARBs), and mineralocorticoid receptor antagonists. ACEIs and ARBs are central to the treatment of hypertension, heart failure with reduced ejection fraction, and chronic kidney disease.^154,155^ One advantage of ARBs over ACEIs is the lower risk of side effects such as cough and angioedema, the latter of which can be life-threatening due to swelling and obstruction of the airway.^156^ In 1987, there was great optimism surrounding ACEIs for their use in congestive heart failure following the CONSENSUS trial.^157^ (Table 1). This study showed that adding enalapril to a conventional heart failure regimen that at the time was typically furosemide and digoxin, reduced mortality by 40% at 6 months and 27% compared to the end of the 12-month study. Following the positive studies and enthusiasm for ACEIs, there was interest that ARBs could block RAAS more effectively compared to ACEIs. In 1997 the ELITE trial, compared the ARB losartan, with the ACEI captopril. There was a 32% reduction in death and hospital admissions for heart failure treated with losartan compared with those treated with captopril.^158^ In addition, losartan was better tolerated compared to captopril, with fewer patients discontinuing therapy. More recently, the combination of the ARB valsartan with a neprilysin inhibitor (sacubitril) has demonstrated superiority to enalapril in the treatment of heart failure with reduced ejection fraction in the PARADIGM-HF trial.^159^

Although ACEIs and ARBs are currently mainstays in the treatment of hypertension and heart failure, AT_1_R β-arrestin biased agonists have a theoretical advantage over ARBs that block both G proteins and β-arrestins.^160,161^ G⍺_q_ appears to mediate the majority of pathological actions of chronic AT_1_R activation in the setting of heart failure and is the primary mechanism that drives the hypertrophic response to pressure overload.^162^ Studies in animal models have documented the positive effects of AT_1_R β-arrestin-biased agonists including improved cardiac output, hemodynamic profile, and preserved renal function while retaining the antihypertensive effects of traditional angiotensin receptor blockers.^163,164^ β-arrestin biased AT_1_R signaling has been shown to increase isolated myocyte contractility and in vivo cardiac contractility,^160,165^ and be necessary for the Frank-Starling law of the heart.^166^ There has been a single clinical trial of the β-arrestin biased ligand TRV0027 in the setting of acute systolic heart failure, where the drug failed to show evidence of a beneficial effect.^167^ However, a range of factors, including the study design and the short duration of treatment could have explained the observed lack of efficacy in acute HF. Additionally, a later post-hoc analysis found TRV027 treatment was associated with reduced all-cause and cardiovascular death^168^. Therefore, the question as to whether β-arrestin biased AT_1_R agonists will live up to their therapeutic promise for chronic HF remains open.^169^

### Incretin hormone receptors

Glucagon-like peptide 1 (GLP-1), and glucose-dependent insulinotropic polypeptide (GIP), are two peptide hormones released by the gut that are responsible for mediating increased insulin secretion by the pancreas in response to an oral glucose load. Their receptors are expressed on the beta cells in pancreatic islets and are also expressed in the heart, vasculature, intestines, kidney, and brain.^170,171^ The GLP-1 receptor (GLP-1R) is G⍺_s_- and G⍺_q_-coupled while the GIP receptor (GIPR) only signals through G⍺_s_. The relative ability of both receptors to signal through G⍺_s_ may be altered during diabetes.^82^ Both receptors can also signal through β-arrestin.^170^

Though GLP-1R agonists were originally developed and approved for glycemic control in the setting of diabetes, incretin receptor agonists have been found to improve a much wider range of health outcomes. A recent meta-analysis focusing on cardiovascular and kidney outcomes in diabetics found that use of GLP-1R agonists improved a number of cardiovascular biomarkers and reduce all-cause major adverse cardiovascular events, a composite outcome including nonfatal myocardial infarction, stroke, and cardiovascular death.^172^ Additionally, in the more recent SELECT^173^ and STEP-HFpEF^174^ clinical trials, the GLP-1R agonist semaglutide improved cardiovascular outcomes in patients with obesity in the absence of diabetes. In the wake of these promising findings, GLP-1 receptor agonists have been recognized as “the breakthrough of the year 2023”.^175^

The mechanism by which GLP-1R agonists promote beneficial cardiovascular outcomes is multifactorial. The treatment of obesity, which increases cardiovascular disease risk through the development of dyslipidemia, type two diabetes (T2D), and hypertension, likely plays a role.^176,177^ Additionally, some of their activities appear to be mediated through direct actions on receptors expressed in the cardiovascular system. For example, GLP-1Rs appear to regulate platelet activation^178,179^, vasodilation^180^, and inflammation.^181^ However, the relative contributions of these mechanisms to cardiovascular outcomes are unclear.^171^

Currently under development and in clinical trials are additional incretin receptor agonists. Here the focus has been on three fronts. First, is the development of small molecule agonists of incretin receptors that have improved oral bioavailability compared to currently existing peptide-based agonists.^182,183^ Second, is the development of triple agonists that have the ability to activate GLP, GIP, and glucagon receptors, and which appear to have greater efficacy for glycemic control and weight loss.^184,185^ Finally is the investigation of the role biased agonism plays in the physiological response to these ligands^186,187^. Physiologically, insulin secretion in pancreatic beta cells in response to incretin stimulation is mediated by G⍺_s_, G⍺_q_, and β-arrestin.^82,188,189^ Recent structural studies have provided some insight into the mechanisms underlying ligand bias at the GLP-1R.^190^ Further research is needed to identify the benefits of biased signaling with respect to the mechanisims of these drugs and their relation to cardiovascular outcomes.

## Emerging Paradigms in GPCR Biology and Human Health

The past century has seen dramatic advancements in our understanding of GPCR biology across multiple scales of biological organization. Current and emerging research paradigms include 1) the structural mechanisms underlying GPCR activation and transducer engagement; 2) the use of these data in approaches to structure-based drug discovery; and 3) location/context-dependent mechanisms underlying GPCR signaling. Here we highlight exciting developments in each of these areas and discuss their future directions.

### Structural features underlying GPCR activation and transducer engagement

#### GPCR Structural Biology

The first structure of a GPCR bound to a diffusible ligand to be solved was the β_2_AR in 2007 by a group led by Brian Kobilka.^191,192^ Four years later, researchers led by the same group achieved an even more substantial accomplishment, a crystal structure of the β_2_AR bound to G⍺_s_ transducer.^193^ This first snapshot of a GPCR bound to a transducer provided tremendous insights into the structural basis of GPCR transmembrane signaling, identifying broad structural rearrangements of both receptor and transducer, and the specific amino acid interactions between the receptor and transducer stabilizing these interactions.

Since the structural determination of the β_2_AR in 2007, the number of solved structures of GPCRs available in the protein data bank (PDB) has increased dramatically. Within a year of solving the β_2_AR, three additional structures of other GPCRs had been solved. By the end of 2023, the number has increased to 180 unique receptors. In all, of the 180 unique receptors, there are over 1,000 structures of GPCRs in varying conformations occupied by different ligands, or coupled to different transducers.^194,195^ Furthermore, computer prediction models such as AlphaFold have the potential to produce models of the over 600 receptors whose structures have not yet been solved experimentally.^196^

Improvements in the structural determination of GPCRs have been driven by several methodological advances. They range from improved detergent systems, stabilizing mutations or fusion proteins, conformation-specific nanobodies, and technical improvements in X-ray crystallography and cryo-EM.^197^ For example, methodological advances necessary for solving the structure of the human β_2_AR included sufficient expression of the β_2_AR through virus-based expression methods and further stabilization by the addition of either a receptor-specific fragment antigen-binding region (Fab) or receptor modifications, such as an engineered T4L lysozyme.^191,192^ Advancements in cryo-EM have sparked a further GPCR structural revolution,^197^ with an increasing number of receptor:transducer complex structures stabilized by nanobodies or Fabs.

#### Towards an understanding of GPCR dynamics

**Figure 5:**
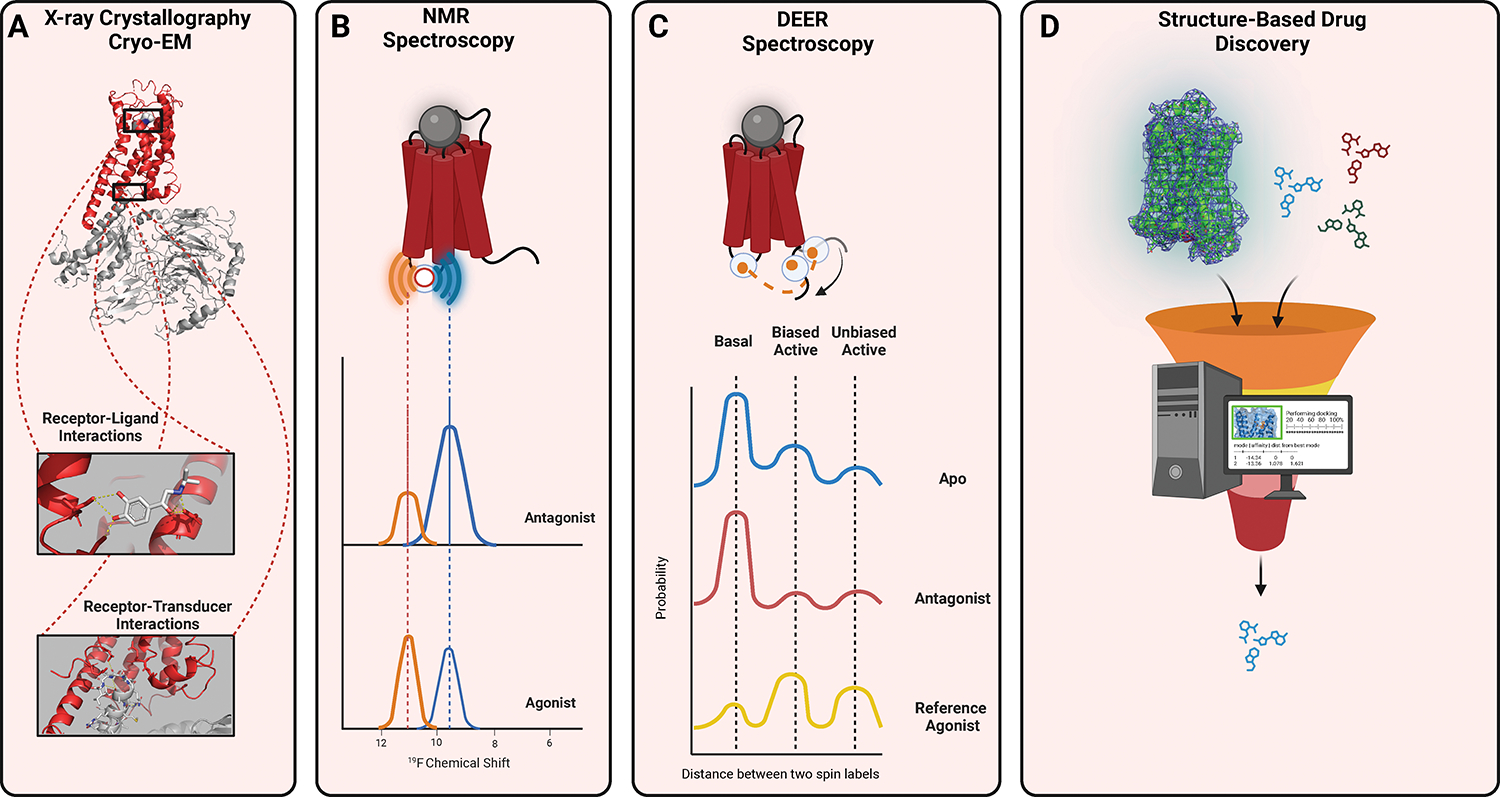
Structural studies of GPCRs. (**A**) X-ray crystallography and CryoEM provide static structures of GPCRs. With improvements in cryoEM technology and workflow, cryoEM can now provide high-resolution data on both receptor-ligand and receptor-transducer interactions, making it particularly well-suited to the study of GPCRs. (**B**) NMR Spectroscopy with probes such as ^19^F can provide information on dynamics and conformational ensembles by measuring changes in the local environment of individually labeled probes. (**C**) EPR spectroscopy techniques such as DEER spectroscopy with two spin-label probes can provide information on distances, conformations, and their relative populations. (**D**) Structure-Based Drug Discovery techniques use receptor structures to allow molecular docking and other approaches to screen compounds that bind to a desired receptor conformation.

Though techniques for obtaining GPCR structures have advanced considerably, a key limitation of these methods is that they can only provide singular snapshots of a GPCR - ones that are highly influenced by the particular crystallization/cryo-EM conditions, and by the artificial presence of stabilizing mutations or antibodies (Figure 5A). Furthermore, as opposed to static structures, receptors are highly dynamic and adopt multiple conformations, with distinct conformations mediating specific signaling outcomes.^121^ As a result, the study of these conformations and their dynamics is essential to our understanding of GPCR biology. Therefore, the ability to characterize the structural differences and dynamics of receptor conformations is paramount. Two methods have come to the fore: nuclear magnetic resonance (NMR) spectroscopy and site-directed spin labeling electron paramagnetic resonance (EPR) spectroscopy (Figure 5B–C).^198,199^ These methods rely on labeling specific residues within receptors and measuring changes in their chemical environment (NMR) or changes in distances from other labeled residues (EPR). The concept of conformational heterogeneity is especially important in the study of biased agonism, as the presence of multiple conformations explains the ability of receptors to interact with distinct transducers with different efficiencies. For example, NMR studies of the β_2_AR occupied by ligands with varying biases have provided evidence of unique conformational states in helix VII associated with β-arrestin-biased states.^200^ Similar conformational studies have been performed on the μ opioid and adenosine receptors.^201,202^ In a landmark study of a panel of AT_1_R-biased agonists, distinct populations of AT_1_R conformations stabilized by biased ligands were uncovered with EPR using a specific EPR technique known as Pulsed EPR double electron–electron resonance (DEER) spectroscopy.^123^

### New Approaches to GPCR Drug Discovery

#### Structure-based Drug Design

The wealth of structural information on GPCR has ushered in a “golden” age of structure-based drug design for GPCRs. The increasing availability of high-quality receptor structures combined with advancements in virtual drug screening have played key roles in this process, and have already been applied to design ligands for numerous receptors.^203,204^ Facilitating this boon of structure-based drug design have been improvements in computational hardware, refined screening/docking algorithms, and optimizations of virtual libraries for screening. For example, a recent virtual screening campaign at the melanocortin 1 receptor was able to screen more than 150 million compounds; the top 300,000 compounds were further sorted and forty representative compounds from the top 0.1% were synthesized.^205,206^ Surprisingly, of the synthesized compounds, 15 compounds showed activity, with a final hit rate of close to 40% using this screening strategy. Novel approaches such as synthon-based ligand discovery allow the virtual screening of billions of compounds.^207^ Despite the success of such screens, there still is room for refinements in pose prediction programs and the ability to evaluate compound binding from the perspective of receptor dynamics.^203,204^ Further challenges of structure-based drug discovery are to tackle the potential binding at multiple allosteric sites and screening for biased agonists (Figure 5D).^208^

#### Allosteric Modulators

**Figure 6:**
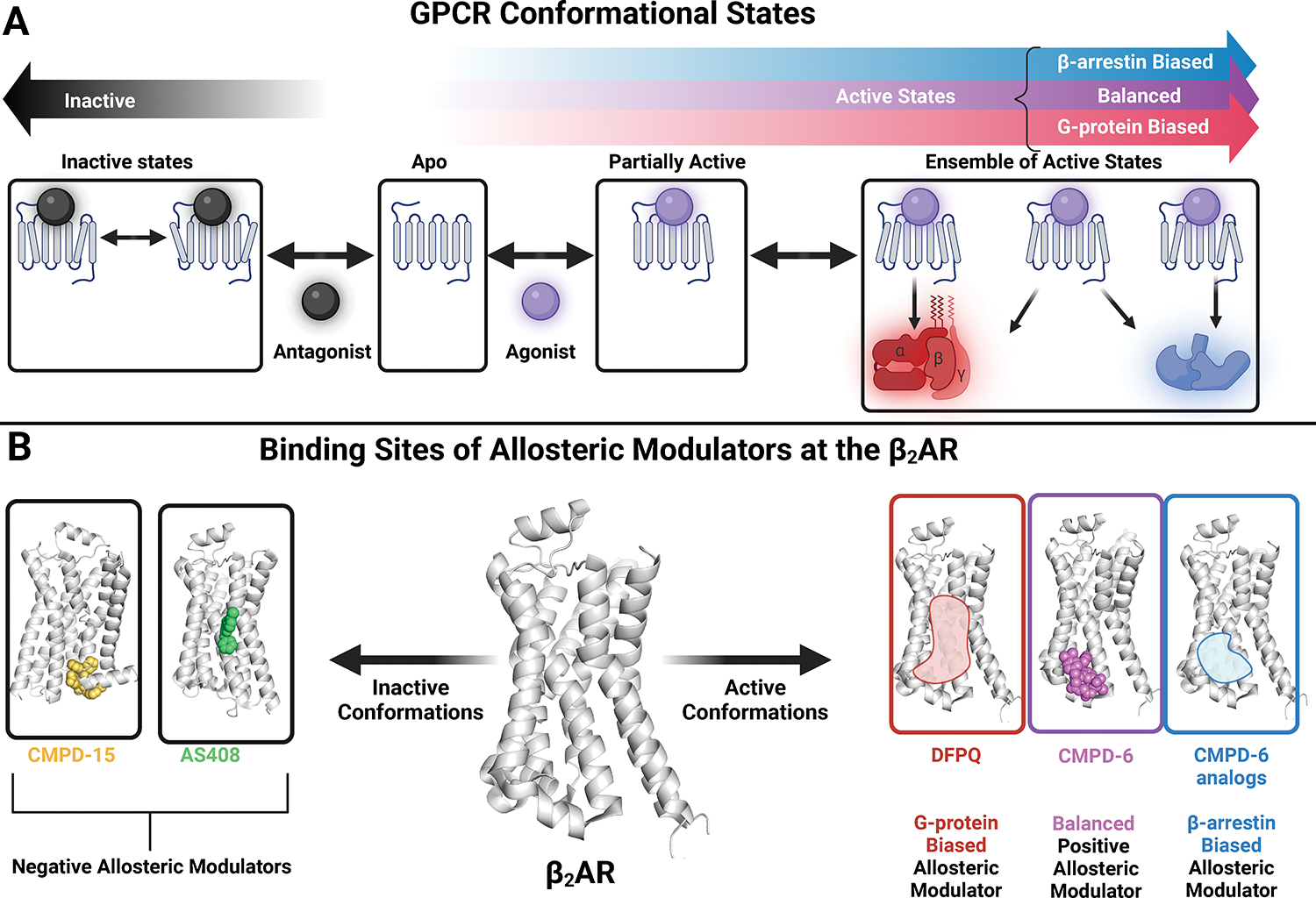
Allosteric Modulation of GPCRs. (**A**) GPCR Conformational States. Receptors in the Apo (no ligands bound) state are largely in inactive conformations but can be stabilized by orthosteric agonists and antagonists in a variety of different active or inactive states. Active states are characterized by an opening of the transducer binding pocket. This pocket is closed to different degrees in inactive states. Orthosteric agonists can activate both G-protein and β-arrestin pathways or be biased towards one pathway. (**B**) Allosteric modulators bind to topographically distinct sites from the orthosteric site that can modulate receptor conformation. Allosteric modulators can either enhance agonist affinity and/or efficacy (positive allosteric modulators) or diminish the agonist affinity and/or efficacy (negative allosteric modulators). In addition, positive allosteric modulators can stabilize specific receptor conformations that promote bias towards G-protein or β-arrestin. Illustrated are the binding sites of allosteric modulators at the β_2_AR and the conformations they promote. The negative allosteric modulators of the β_2_AR, CMPD-15, and AS408, bind to distinct sites on the β_2_AR. CMPD-6, a balanced positive allosteric modulator, binds to another distinct site. Allosteric modulators can also display biased properties such as difluorophenyl quinazoline derivatives (DFPQ) which biases the β_2_AR towards G-protein signaling. The binding pose of DFPQ compounds has not been experimentally solved, but they are believed to bind close to the CMPD-6 binding site. β-arrestin biased analogs of CMPD-6 have also been proposed due to CMPD-6’s interactions with the β-arrestin biased orthosteric ligand carvedilol. Compound structures obtained from the Protein Data Bank (PDB) : CMPD-15: 5X7D, AS408: 60BA, CMPD-6: 6N48.

Traditional GPCR-targeted drug development has focused primarily on ligands that bind to the receptor’s extracellular facing orthosteric ligand binding site. However, receptor activation is linked to conformational changes across the entire receptor, with the existence of multiple conformationally distinct inactive and active states^209^ (Figure 6A). As a result, the receptor surface contains additional sites that can be targeted by *allosteric* modulators. These allosteric modulators often bind nearby key activation switches on the receptor surface such as the conserved aspartic acid-arginine-tyrosine (DRY) motif, and stabilize specific interactions, promoting particular conformations of the receptor.^210,211^ Allosteric modulators also frequently display complex pharmacology. Because allosteric modulators bind at distinct sites from orthosteric ligands, they can affect the potency of orthosteric ligands as well as their signaling efficacy. Additionally, they may display “probe dependence,” where the effects of an allosteric modulator may differ depending on the specific orthosteric ligand bound to the receptor.^140^ Allosteric modulators can be defined as positive (enhance orthosteric agonist binding/activity), negative (diminish it), silent (no effect on the orthosteric agonist), or biased (promote a biased response to an orthosteric agonist).^212^

At the β_2_AR, allosteric modulators have been found to bind to at least three separate sites and promote distinct active or inactive conformations of the receptor, showcasing the druggability of diverse allosteric sites and the manifold modulatory effects associated with them^210,211,213^ For example, the negative allosteric modulator AS408 binds between transmembrane helix 3 and 5 of the β_2_AR, stabilizing key residues in an inactive conformation.^211^ On the other hand, the negative allosteric modulator CMPD-15 binds to an allosteric site in the intracellular surface of the β_2_AR. There, in addition to stabilizing an inactive conformation of the receptor, it also physically interferes with transducer binding, resulting in a dual mechanism of receptor inhibition.^213^

There is also one solved structure of a positive allosteric modulator bound to the β_2_AR stabilizing an active conformation capable of signaling through both G-protein and β-arrestin.^210^ CMPD-6 binds to the base of transmembrane helix 3 and 5 and promotes an active conformation by stabilizing key activation switches, including the formation of a helix within intracellular loop 2, and stabilizing a conserved DRY motif found in many GPCRs.^210^ Interestingly, CMPD-6 also displays unique cooperativity with the arrestin biased ligand carvedilol, suggesting that analogs selectively biased towards potentiating β-arrestin signaling could be developed for this site as well.^140^ Besides CMPD-6, additional allosteric modulators with biased properties have been identified for the β_2_AR. These include DFPQ derivatives, isolated from high throughput screening in cells, and CMPD-36, 37, and 42, isolated from computational screening of allosteric ligands for the β_2_AR.^214,215^ In general these compounds inhibit β-arrestin signaling by orthosteric ligands while permitting G-protein signaling, thus biasing receptor signaling towards G-protein. Computational docking and mutagenesis studies suggest that these compounds bind to a range of allosteric sites on the β_2_AR. However, their structures have not yet been solved, so the key molecular switches they stabilize are yet to be determined (Figure 6B).

In contrast to the orthosteric site, which is well-defined with respect to its location and ligand binding, allosteric sites across the receptor surface vary greatly. Due to their diversity, allosteric modulators offer several advantages over traditional orthosteric drugs. First, allosteric sites often have the opportunity to encode greater receptor subtype specificity. Many receptors for the same agonist, e.g., adrenergic receptors, have tight evolutionary constraints on the orthosteric site, so drugs targeting that site will likely bind to multiple receptor subtypes. As a result of the reduced evolutionary constraints at allosteric sites, which do not need to maintain specificity for binding to the endogenous orthosteric ligand, allosteric modulators may be designed to yield subtype-specific drugs. Additionally, allosteric modulators can regulate the binding and signaling efficacy of orthosteric ligands in multiple ways, achieving signaling outcomes that would not be possible by targeting the orthosteric site alone. This last point is particularly important: as a consequence of binding to sites on the receptor distinct from the orthosteric site, allosteric modulators may be able to stabilize biased receptor signaling conformations that may be difficult or impossible to achieve with an orthosteric ligand.^208^ For example, a β-arrestin-biased allosteric modulator for the neurotensin receptor, ML314, and its derivative, SBI-553, selectively antagonize G-protein signaling while attenuating addictive behaviors via β-arrestin-dependent signaling processes.^216^ This relies on its ability to sterically interfere with G⍺_q_-protein coupling, but not coupling to GRK or β-arrestin.^217,218^

### Signaling from Subcellular Compartments

#### Nanodomains

**Figure 7:**
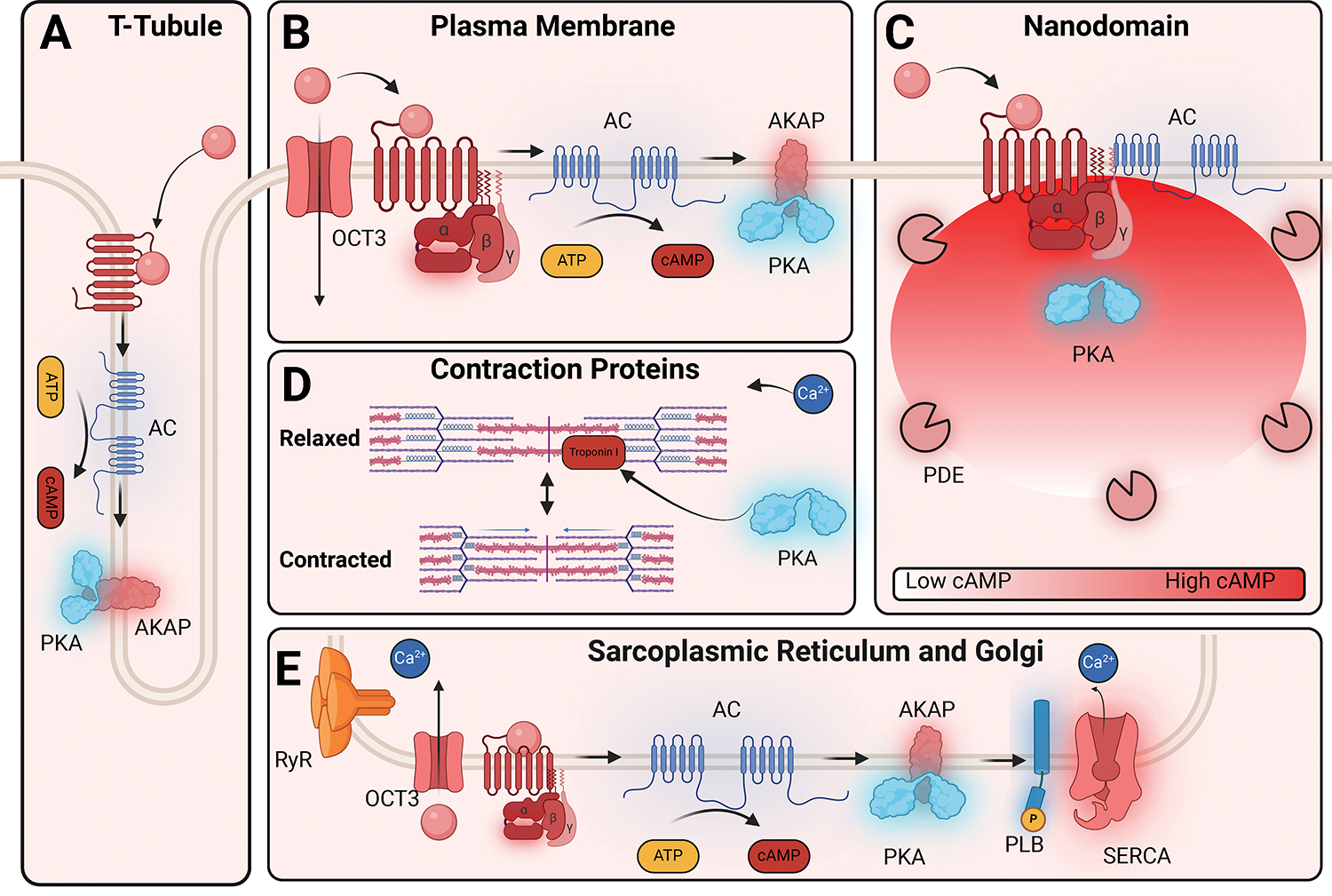
Location Bias in Cardiac Myocytes. (**A**) In healthy cardiac tissue, βARs are found in T-tubules, which when stimulated with epinephrine/norepinephrine activate βAR-AC-PKA. PKA is tethered to intracellular compartments by AKAPs. PKA phosphorylates the RyR, inducing calcium release and resulting in increased cardiac contractility. (**B**) βARs at the plasma membrane enhance contractility through βAR-AC-PKA activation. (**C**) Recent work has evaluated cAMP nanodomains with different Gα_s_-mediated receptors. Nanodomains are membraneless compartments that enhance or sequester signaling molecules. For example, PDE is involved in regulating the size and shape of cAMP nanodomains, whereas AKAP (AKAP not pictured) serves as a PKA scaffolding protein to sequester cAMP signaling. (**D**) Contractility is promoted by Ca^2+^ binding to troponin C in addition to PKA mediated-phosphorylation of contractile proteins such as troponin I (**E**) OCT3 is found on the plasma membrane and the Golgi and facilitates the transportation of norepinephrine/epinephrine into the cell to promote Golgi-βARs-AC-PKA signaling. Unlike the pool of PKA found at the plasma membrane and T-tubules, Golgi PKA activates phospholamban and forms an inhibitory complex with SERCA. This reduces available Ca^2+^ and increases the rate of relaxation. βARs, β-adrenergic receptor; AC, adenyl cyclase; ATP, adenosine triphosphate; cAMP, cyclic adenosine monophosphate; PKA; Protein kinase A; AKAP; A-kinase anchoring protein; PDE, phosphodiesterase; PLB, phospholamban; SERCA, Sarcoendoplasmic Reticulum Calcium ATPase; OCT3, monoamine transporter; RyR, Ryanodine Receptor

There is increasing evidence that signals generated by receptors are confined to nanometer-sized domains, referred to as nanodomains (Figure 7).^219^ For example, functionally divergent pools of βARs contribute to cAMP compartmentalization to fine-tune physiological cardiac functions such as contractility. Early work in the 1980s proposed that cAMP microdomains are regulated by tethered PKA in cardiomyocytes (Figure 7C).^220^ With the development of cAMP and EPAC fluorescence resonance energy transfer (FRET)-based biosensors, monitoring second messengers in subcellular locations became possible. Cardiac myocytes transfected with PKA FRET biosensors localized to the sarcoplasmic reticular and myofilament sites revealed heterogeneity in kinetics and amplitude of cAMP signaling.^221^ cAMP microdomains have been shown to influence cardiac contractility and dysregulation of these domains has been implicated in heart failure (Figure 7D).^222^ Recently, engineered nanoruler FRET biosensors have identified cAMP nanodomains at the GLP-1R and β_2_AR, suggesting that these domains are the fundamental units of signaling in the cell.^223^ These cAMP domains are dynamic entities, localized to specific subcellular regions with tight regulation of cAMP levels that are distinct from bulk cytosolic cAMP. In general, these domains are regulated by AC, phosphodiesterases (which break down cAMP), and scaffold proteins such as A-kinase anchoring proteins (AKAP), which contribute to this localization.

#### Location bias

Not long after the discovery that GPCRs could signal from distinct nanodomains, it was realized that this signaling could differ between subcellular compartments, a phenomenon now commonly referred to as location bias. It is now appreciated that many receptors, such as β_1_AR, β_2_AR, and AT_1_R, display location bias from different subcellular locations such as the nucleus, endosomes, and Golgi, which result in physiologically distinct outcomes.^224–226^ In 1998, the first evidence of receptor internalization being required for MAPK activation was demonstrated at the β_2_AR in the Lefkowitz Lab. In that study, it was shown that expressing β-arrestin or dynamin dominant negative mutants in HEK293 cells inhibited MAPK activation but did not limit the receptor’s ability to couple to G proteins.^227^ Subsequent work supported that receptor internalization did not simply terminate signaling, but promoted signaling by G-proteins from endosomes.^228^ In 2009, three independent groups showed that thyroid stimulating hormone, parathyroid hormone, and the sphingosine-1 phosphate receptors continue to signal after their internalization.^229–231^ Inhibition of internalization resulted in the ablation of cAMP signaling, demonstrating that G protein signaling can occur from endosomes. Subsequent work showed that other G⍺_s_-coupled receptors, such as internalized β_2_ARs, increase intracellular cAMP levels, which was subsequently shown to promote CREB-dependent transcription.^224,232^

A few studies have probed the consequences of location-biased signaling in physiologically relevant cell types, such as cardiomyocytes, in health and disease.^222^ β_1_ARs are found throughout the cardiomyocyte, while the β_2_AR and β_3_AR subtypes are solely expressed in the T-tubules,^233^ and cAMP generation from receptors at T-tubules activates a subset of PKA anchored in their vicinity^234^ (Figure 7A). Receptors are localized to other subcellular compartments such as the nuclear membrane, where α_1_ARs,^235,236^ AT_1_Rs,^237^ ETARs,^238^ and βARs^129^ are present in cardiomyocytes. Nuclear α_1_AR translocates from the nucleus to the caveolae to induce ERK signaling ^129,235^ and AT_1_Rs in the nucleus has been observed to regulate N-κB transcription.^237^ β_1_ARs and β_2_ARs are also found on nuclear membranes of cardiomyocytes where they activate Gα_s_ and other signaling partners, which alter transcriptional networks upon receptor activation.^129^ Another important subcellular compartment is the Golgi/sarcoplasmic reticulum, where a pool of β_1_ARs resides.^225^ The accessibility of different ligands to this pool of receptors depends on their membrane permeability, which can result in distinct physiological responses. The organic cation transporter subtype 3 (OCT3), has been shown to facilitate the uptake of membrane-impermeable catecholamines across the plasma membrane to the nucleus and Golgi.^235^ For example, OCT3-mediated transported ligands can regulate the sarcoplasmic reticulum localized β_1_AR and regulate contractility through local PKA-mediated phosphorylation of phospholamban (Figure 7E). The function of β_1_AR localization has been further elucidated in intact zebrafish hearts, where Golgi-localized β_1_AR cAMP production promotes lusitropy (through PLB/SERCA), while plasma membrane-localized β_1_AR mediates PKA phosphorylation of RyR2 and troponin I to promote inotropy.^239^ Further work with drugs with different membrane permeability may aid in uncovering the physiological significance of subcellular signaling in cardiomyocytes.^240^

### Conclusions

Our understanding of cardiovascular GPCRs has had a profound impact on the development of modern-day cardiovascular therapies. Over the past century, researchers have made many key discoveries on the nature of GPCR signaling, elucidating multiple mechanisms of drug action. With the substantial growth and excitement around cardiovascular GPCRs, these receptors are still some of the top targets in drug discovery. Additional understanding of receptor structures and receptor dynamics, will improve screening approaches, including those that target new allosteric sites on receptors and incorporate novel computational and artificial intelligence approaches. These could also be used to guide the development of drugs that have bias between heterotrimeric G-proteins and β-arrestins as well as having specific patterns of location bias. With new data sets, such as those from single-cell sequencing and other approaches, it should also be possible to identify novel therapeutic targets for cardiovascular disease. Combining these approaches, across pharmacology, physiology, structure, and computational biology, should lead to an exciting century-to-come of new GPCR-targeting therapies in cardiovascular disease.

## Figures and Tables

**Table 1.** Notable FDA-approved drugs, physiological effects, and cardiovascular clinical trials of drugs targeting the β-adrenergic, angiotensin II type 1, and incretin receptors.

Receptor Activity	Proximal Effectors	FDA-approved Agents	Cardiovascular Effects	Notable Clinical Trials
β-adrenergic receptors (β_1_AR and β_2_AR) antagonists	Gα_s_, Gα_i_, β -arrestin 1/2	**Beta blockers:** Acebutolol, Atenolol, Betaxolol, Bisoprolol, Carvedilol, Labetalol, Metoprolol, Nadolol, Nebivolol, Pindolol, Propranolol, Timolol	- Heart rate control - Negative inotropy - Reduction in myocardial oxygen demand - Reduces BP - Treatment of heart failure	**Heart Failure with Reduced Ejection Fraction:** 1993: Metoprolol in Dilated Cardiomyopathy (*Metoprolol*)^53^ 1996: US Carvedilol HF Study (Carvedilol)^54^ 1999: MERIT-HF (Metoprolol)^56^ 1999: CIBIS-II (Bisoprolol)^55^ 2001: COPERNICUS (Carvedilol)^57^
Angiotensin II Type 1 Receptor (AT_1_R) antagonists	Gα_q_, Gα_i_, β -arrestin 1/2	**ACE-I:** Benazepril, Captopril, Enalapril, Fosinopril, Lisinopril, Moexipril, Perindopril, Quinapril, Ramipril, Transolapril **ARB:** Candesartan, Eprosartan, Irbesartan, Losartan, Olmesartan, Telmisartan, Valsartan	- Reduces BP - Treatment of heart failure - Renoprotection by decreasing glomerular filtration	**Heart Failure with Reduced Ejection Fraction:** 1987: CONSENSUS (Enalapril)^157^ 1991: V-HeFT II (Enalapril)^252^ 1991: SOLVD-T (Enalapril)^253^ 1992: SOLVD-P (Enalapril)^254^ 1997: ELITE (Losartan / Captopril)^158^ 2001: Val-HeFT (Valsartan)^255^ 2003: CHARM-Alt (Candesartan)^256^ 2014: PARADIGM-HF (Sacubitril-Valsartan / Enalapril)^159^
Glucagon-like peptide 1 receptor (GLP-1R) agonists	Gα_s_, Gα_q_, Gα_i_, β -arrestin 1	Dulaglutide, Exenatide, Liraglutide, Lixisenatide, Tirzepatide (dual GLP-1R and GIPR agonist)	- Weight loss - Glycemic control - Reduction in major cardiovascular events - Improvement in heart failure	**Major adverse cardiovascular events (Nonfatal myocardial infarction, nonfatal stroke, or cardiovascular death):** 2021 Meta-analysis of eight clinical trials in diabetes (Lixisenatide, Liraglutide, Semaglutide, Exenatide, Albiglutide, Dulaglutide, Efpeglenatide)^172^ 2023 SELECT – Obesity and CAD without diabetes (Semaglutide)^173^ **Heart Failure with Preserved Ejection Fraction:** 2023 STEP-HFpEF – Obesity without diabetes (Semaglutide)^174^

## Non-standard Abbreviations and Acronyms

GPCR: G protein-coupled receptor
cAMP: cyclic adenosine monophosphate
AC: adenylyl cyclase
GTP: guanosine triphosphate
GDP: guanosine diphosphate
βAR: β-adrenergic receptor
GRK: GPCR kinases
AT1R: angiotensin II type 1 receptor
HF: heart failure
ATP: adenosine triphosphate
PKA: protein kinase A
PIP_2_: phosphatidylinositol 4,5-bisphosphate
IP_3_: inositol 1,4,5-trisphosphate
DAG: diacylglycerol
SR: sarcoplasmic reticulum
PKC: protein kinase C
RGS: regulators of G protein signaling
GAPs: GTPase activating proteins
GLP-1: Glucagon-like peptide-1
GLP-1R: The GLP-1 receptor
GIP: glucose-dependent insulinotropic polypeptide
GIPR: GIP receptor
PDB: protein data bank
Fab: fragment antigen-binding
NMR: nuclear magnetic resonance
EPR: electron paramagnetic resonance
DEER: double electron–electron resonance
DRY: aspartic acid-arginine-tyrosine
FRET: fluorescence resonance energy transfer
OCT3: organic cation transporter subtype 3
PLC- β: phospholipase C- β
ERK: extracellular signaling-related kinases
AKAP: A-kinase anchoring protein
PDE: phosphodiesterase
PLB: hospholamban
SERCA: Sarcoendoplasmic Reticulum Calcium ATPase

## References

[1] Hauser AS, Attwood MM, Rask-Andersen M, Schioth HB, Gloriam DE. Trends in GPCR drug discovery: new agents, targets and indications. Nat Rev Drug Discov. 2017;16:829–842. doi: 10.1038/nrd.2017.178 29075003 PMC6882681

[2] Insel PA, Sriram K, Gorr MW, Wiley SZ, Michkov A, Salmerón C, Chinn AM. GPCRomics: an approach to discover GPCR drug targets. Trends Pharmacol Sci. 2019;40:378–387. doi: 10.1016/j.tips.2019.04.001 31078319 PMC6604616

[3] Chen KK, Schmidt CF. The action and clinical use of ephedrine: an alkaloid isolated from the Chinese drug Ma Huang. JAMA. 1926;87:836–842. doi: 10.1001/jama.1926.02680110036011 13809594

[4] Takamine J Adrenalin the active principle of the suprarenal glands and its mode of preparation. American Journal of Pharmacy (1835–1907). 1901:523.

[5] Abel Parascandola J., Takamine, and the isolation of epinephrine. J Allergy Clin Immunol. 2010;125:514–517. doi: 10.1016/j.jaci.2009.11.044 20196206

[6] Arthur G Epinephrine: a short history. The Lancet Respiratory Medicine. 2015;3:350–351. doi: 10.1016/s2213-2600(15)00087-9 25969360

[7] Ehrlich P Croonian Lecture: on immunity with special reference to cell life. Proceedings of the Royal Society of London. 1899;66:424–448.

[8] Drews J Paul Ehrlich: Magister Mundi. Nat Rev Drug Discov. 2004;3:797–801. doi: 10.1038/nrd1498 15340389

[9] Langley JN. On the reaction of cells and of nerve-endings to certain poisons, chiefly as regards the reaction of striated muscle to nicotine and to curari. J Physiol. 1905;33:374–413. doi: 10.1113/jphysiol.1905.sp001128 16992819 PMC1465797

[10] Dale H Modes of drug action. General introductory address. Transactions of the Faraday Society. 1943;39:319b. doi: 10.1039/tf943390319b

[11] Ahlquist RP. Adrenergic receptors: a personal and practical view. Perspect Biol Med. 1973;17:119–122. doi: 10.1353/pbm.1973.0047 4148041

[12] Goodman LS, Gilman A. The pharmacological basis of therapeutics; a textbook of pharmacology, toxicology, and therapeutics for physicians and medical students. 2d ed. New York,: Macmillan; 1955.

[13] Colquhoun D The quantitative analysis of drug-receptor interactions: a short history. Trends Pharmacol Sci. 2006;27:149–157. doi: 10.1016/j.tips.2006.01.008 16483674

[14] Rang HP. The receptor concept: pharmacology’s big idea. Br J Pharmacol. 2006;147 Suppl 1:S9–16. doi: 10.1038/sj.bjp.0706457 16402126 PMC1760743

[15] Hill AV. The mode of action of nicotine and curari, determined by the form of the contraction curve and the method of temperature coefficients. J Physiol. 1909;39:361–373. doi: 10.1113/jphysiol.1909.sp001344 16992989 PMC1533665

[16] Clark AJ. The reaction between acetyl choline and muscle cells. J Physiol. 1926;61:530–546. doi: 10.1113/jphysiol.1926.sp002314 16993813 PMC1514867

[17] Gaddum JH. The quantitative effects of antagonistic drugs. J Physiol. 1937;89:7P–9P.

[18] Schild H pA, a new scale for the measurement of drug antagonism. Br J Pharmacol Chemother. 1947;2:189. 20258355 10.1111/j.1476-5381.1947.tb00336.x PMC1509780

[19] Ariens EJ. Affinity and intrinsic activity in the theory of competitive inhibition. I. Problems and theory. Archives internationales de pharmacodynamie et de therapie. 1954;99:32–49. 13229418

[20] Stephenson RP. A modification of receptor theory. Br J Pharmacol Chemother. 1956;11:379–393. 13383117 10.1111/j.1476-5381.1956.tb00006.x PMC1510558

[21] Sutherland EW, Rall TW. Fractionation and characterization of a cyclic adenine ribonucleotide formed by tissue particles. J Biol Chem. 1958;232:1077–1091. 13549488

[22] Sutherland EW, Rall TW, Menon T. Adenyl Cyclase. J Biol Chem. 1962;237:1220–1227. doi: 10.1016/s0021-9258(18)60312-6 13918525

[23] Rall TW, Sutherland EW. Adenyl cyclase. II. The enzymatically catalyzed formation of adenosine 3’,5’-phosphate and inorganic pyrophosphate from adenosine triphosphate. J Biol Chem. 1962;237:1228–1232. 14490038

[24] Rodbell M, Birnbaumer L, Pohl SL, Krans HM. The glucagon-sensitive adenyl cyclase system in plasma membranes of rat liver. V. An obligatory role of guanylnucleotides in glucagon action. J Biol Chem. 1971;246:1877–1882. 4926550

[25] Rodbell M, Krans HM, Pohl SL, Birnbaumer L. The glucagon-sensitive adenyl cyclase system in plasma membranes of rat liver. IV. Effects of guanylnucleotides on binding of 125I-glucagon. J Biol Chem. 1971;246:1872–1876. 4993962

[26] Rodbell M, Lin MC, Salomon Y. Evidence for interdependent action of glucagon and nucleotides on the hepatic adenylate cyclase system. J Biol Chem. 1974;249:59–65. 4358641

[27] Ross EM, Gilman AG. Resolution of some components of adenylate cyclase necessary for catalytic activity. J Biol Chem. 1977;252:6966–6969. 903346

[28] Northup JK, Sternweis PC, Smigel MD, Schleifer LS, Ross EM, Gilman AG. Purification of the regulatory component of adenylate cyclase. Proc Natl Acad Sci U S A. 1980;77:6516–6520. doi: 10.1073/pnas.77.11.6516 6935665 PMC350316

[29] Black JW, Crowther AF, Shanks RG, Smith LH, Dornhorst AC. A new adrenergic betareceptor antagonist. Lancet. 1964;1:1080–1081. doi: 10.1016/s0140-6736(64)91275-9 14132613

[30] Quirke V Putting theory into practice: James Black, receptor theory and the development of the beta-blockers at ICI, 1958–1978. Med Hist. 2006;50:69–92. doi: 10.1017/s0025727300009455 16502872 PMC1369014

[31] Levitzki A, Atlas D, Steer ML. The binding characteristics and number of beta-adrenergic receptors on the turkey erythrocyte. Proc Natl Acad Sci U S A. 1974;71:2773–2776. doi: 10.1073/pnas.71.7.2773 4528016 PMC388553

[32] Lefkowitz RJ, Mukherjee C, Coverstone M, Caron MG. Stereospecific (3H)(minus)-alprenolol binding sites, beta-adrenergic receptors and adenylate cyclase. Biochem Biophys Res Commun. 1974;60:703–709. doi: 10.1016/0006-291x(74)90297-6 4370935

[33] Aurbach GD, Fedak SA, Woodard CJ, Palmer JS, Hauser D, Troxler F. Beta-adrenergic receptor: stereospecific interaction of iodinated beta-blocking agent with high affinity site. Science. 1974;186:1223–1224. doi: 10.1126/science.186.4170.1223 4154497

[34] Lefkowitz RJ, Mullikin D, Caron MG. Regulation of beta-adrenergic receptors by guanyl-5’-yl imidodiphosphate and other purine nucleotides. J Biol Chem. 1976;251:4686–4692. 947904

[35] De Lean A, Stadel JM, Lefkowitz RJ. A ternary complex model explains the agonist-specific binding properties of the adenylate cyclase-coupled beta-adrenergic receptor. J Biol Chem. 1980;255:7108–7117. 6248546

[36] Caron MG, Srinivasan Y, Pitha J, Kociolek K, Lefkowitz RJ. Affinity chromatography of the beta-adrenergic receptor. J Biol Chem. 1979;254:2923–2927. 218957

[37] Dixon RA, Kobilka BK, Strader DJ, Benovic JL, Dohlman HG, Frielle T, Bolanowski MA, Bennett CD, Rands E, Diehl RE, Cloning of the gene and cDNA for mammalian beta-adrenergic receptor and homology with rhodopsin. Nature. 1986;321:75–79. doi: 10.1038/321075a0 3010132

[38] Benovic JL, Strasser RH, Caron MG, Lefkowitz RJ. Beta-adrenergic receptor kinase: identification of a novel protein kinase that phosphorylates the agonist-occupied form of the receptor. Proc Natl Acad Sci U S A. 1986;83:2797–2801. doi: 10.1073/pnas.83.9.2797 2871555 PMC323393

[39] Lohse MJ, Benovic JL, Codina J, Caron MG, Lefkowitz RJ. Beta-arrestin: a protein that regulates beta-adrenergic receptor function. Science. 1990;248:1547–1550. 2163110 10.1126/science.2163110

[40] Attramadal H, Arriza JL, Aoki C, Dawson TM, Codina J, Kwatra MM, Snyder SH, Caron MG, Lefkowitz RJ. Beta-arrestin2, a novel member of the arrestin/beta-arrestin gene family. J Biol Chem. 1992;267:17882–17890. 1517224

[41] Benovic JL, Kuhn H, Weyand I, Codina J, Caron MG, Lefkowitz RJ. Functional desensitization of the isolated beta-adrenergic receptor by the beta-adrenergic receptor kinase: potential role of an analog of the retinal protein arrestin (48-kDa protein). Proc Natl Acad Sci U S A. 1987;84:8879–8882. 2827157 10.1073/pnas.84.24.8879 PMC299654

[42] Laporte SA, Oakley RH, Zhang J, Holt JA, Ferguson SS, Caron MG, Barak LS. The beta2-adrenergic receptor/betaarrestin complex recruits the clathrin adaptor AP-2 during endocytosis. Proc Natl Acad Sci U S A. 1999;96:3712–3717. 10097102 10.1073/pnas.96.7.3712 PMC22359

[43] Goodman OB Jr, Krupnick JG, Santini F, Gurevich VV, Penn RB, Gagnon AW, Keen JH, Benovic JL. Beta-arrestin acts as a clathrin adaptor in endocytosis of the beta2-adrenergic receptor. Nature. 1996;383:447–450. doi: 10.1038/383447a0 8837779

[44] Luttrell LM, Ferguson SS, Daaka Y, Miller WE, Maudsley S, Della Rocca GJ, Lin F, Kawakatsu H, Owada K, Luttrell DK, Beta-arrestin-dependent formation of beta2 adrenergic receptor-Src protein kinase complexes. Science. 1999;283:655–661. doi: 10.1126/science.283.5402.655 9924018

[45] Bristow MR, Ginsburg R, Umans V, Fowler M, Minobe W, Rasmussen R, Zera P, Menlove R, Shah P, Jamieson S. Beta 1-and beta 2-adrenergic-receptor subpopulations in nonfailing and failing human ventricular myocardium: coupling of both receptor subtypes to muscle contraction and selective beta 1-receptor down-regulation in heart failure. Circ Res. 1986;59:297–309. 2876788 10.1161/01.res.59.3.297

[46] Myagmar BE, Flynn JM, Cowley PM, Swigart PM, Montgomery MD, Thai K, Nair D, Gupta R, Deng DX, Hosoda C, Adrenergic receptors in individual ventricular myocytes: The beta-1 and alpha-1B are in all cells, the alpha-1A Is in a subpopulation, and the beta-2 and beta-3 are mostly absent. Circ Res. 2017;120:1103–1115. doi: 10.1161/circresaha.117.310520 28219977 PMC5376223

[47] Bristow MR, Ginsburg R, Minobe W, Cubicciotti RS, Sageman WS, Lurie K, Billingham ME, Harrison DC, Stinson EB. Decreased catecholamine sensitivity and beta-adrenergic-receptor density in failing human hearts. N Engl J Med. 1982;307:205–211. doi: 10.1056/NEJM198207223070401 6283349

[48] Ungerer M, Böhm M, Elce JS, Erdmann E, Lohse MJ. Altered expression of beta-adrenergic receptor kinase and beta 1-adrenergic receptors in the failing human heart. Circulation. 1993;87:454–463. doi: 10.1161/01.cir.87.2.454 8381058

[49] Cohn JN, Levine TB, Olivari MT, Garberg V, Lura D, Francis GS, Simon AB, Rector T. Plasma norepinephrine as a guide to prognosis in patients with chronic congestive heart failure. N Engl J Med. 1984;311:819–823. doi: 10.1056/NEJM198409273111303 6382011

[50] Engelhardt S, Hein L, Wiesmann F, Lohse MJ. Progressive hypertrophy and heart failure in β1-adrenergic receptor transgenic mice. Proc Natl Acad Sci U S A. 1999;96:7059–7064. 10359838 10.1073/pnas.96.12.7059 PMC22055

[51] Iwase M, Bishop SP, Uechi M, Vatner DE, Shannon RP, Kudej RK, Wight DC, Wagner TE, Ishikawa Y, Homcy CJ. Adverse effects of chronic endogenous sympathetic drive induced by cardiac Gsα overexpression. Circ Res. 1996;78:517–524. 8635208 10.1161/01.res.78.4.517

[52] Milano CA, Allen LF, Rockman HA, Dolber PC, McMinn TR, Chien KR, Johnson TD, Bond RA, Lefkowitz RJ. Enhanced myocardial function in transgenic mice overexpressing the beta 2-adrenergic receptor. Science. 1994;264:582–586. doi: 10.1126/science.8160017 8160017

[53] Waagstein F, Bristow MR, Swedberg K, Camerini F, Fowler MB, Silver MA, Gilbert EM, Johnson MR, Goss FG, Hjalmarson A. Beneficial effects of metoprolol in idiopathic dilated cardiomyopathy. Metoprolol in Dilated Cardiomyopathy (MDC) Trial Study Group. Lancet. 1993;342:1441–1446. doi: 10.1016/0140-6736(93)92930-r 7902479

[54] Packer M, Bristow MR, Cohn JN, Colucci WS, Fowler MB, Gilbert EM, Shusterman NH. The effect of carvedilol on morbidity and mortality in patients with chronic heart failure. U.S. Carvedilol Heart Failure Study Group. N Engl J Med. 1996;334:1349–1355. doi: 10.1056/nejm199605233342101 8614419

[55] The Cardiac Insufficiency Bisoprolol Study II (CIBIS-II): a randomised trial. Lancet. 1999;353:9–13. 10023943

[56] Effect of metoprolol CR/XL in chronic heart failure: Metoprolol CR/XL Randomised Intervention Trial in Congestive Heart Failure (MERIT-HF). Lancet. 1999;353:2001–2007. 10376614

[57] Packer M, Coats AJ, Fowler MB, Katus HA, Krum H, Mohacsi P, Rouleau JL, Tendera M, Castaigne A, Roecker EB, Effect of carvedilol on survival in severe chronic heart failure. N Engl J Med. 2001;344:1651–1658. doi: 10.1056/nejm200105313442201 11386263

[58] Xamoterol in severe heart failure. The Xamoterol in Severe Heart Failure Study Group. Lancet. 1990;336:1–6. 1694945

[59] Witchitz S, Cohen-Solal A, Dartois N, Weisslinger N, Juste K, Darmon JY. Treatment of heart failure with celiprolol, a cardioselective beta blocker with beta-2 agonist vasodilatory properties. The CELICARD Group. Am J Cardiol. 2000;85:1467–1471. doi: 10.1016/s0002-9149(00)00796-7 10856394

[60] Lefkowitz RJ, Rockman HA, Shim PJ, Liu S, Ahn S, Pani B, Rajagopal S, Shenoy SK, Bouvier M, Benovic JL, How carvedilol does not activate β(2)-adrenoceptors. Nat Commun. 2023;14:7866. doi: 10.1038/s41467-023-42848-5 38036531 PMC10689753

[61] Jiang H, Galtes D, Wang J, Rockman HA. G protein-coupled receptor signaling: transducers and effectors. American Journal of Physiology-Cell Physiology. 2022;323:C731–C748. doi: 10.1152/ajpcell.00210.2022 35816644 PMC9448338

[62] Gilman AG. G proteins and dual control of adenylate cyclase. Cell. 1984;36:577–579. doi: 10.1016/0092-8674(84)90336-2 6321035

[63] Papa A, Kushner J, Marx SO. Adrenergic regulation of calcium channels in the heart. Annual Review of Physiology. 2022;84:285–306. doi: 10.1146/annurev-physiol-060121-041653 PMC9573788 34752709

[64] Cheng H, Lederer WJ, Cannell MB. Calcium sparks: elementary events underlying excitation-contraction coupling in heart muscle. Science. 1993;262:740–744. doi: 10.1126/science.8235594 8235594

[65] Hennis K, Biel M, Fenske S, Wahl-Schott C. Paradigm shift: new concepts for HCN4 function in cardiac pacemaking. Pflugers Arch. 2022;474:649–663. doi: 10.1007/s00424-022-02698-4 35556164 PMC9192375

[66] Nakayama H, Bodi I, Maillet M, DeSantiago J, Domeier TL, Mikoshiba K, Lorenz JN, Blatter LA, Bers DM, Molkentin JD. The IP3 receptor regulates cardiac hypertrophy in response to select stimuli. Circ Res. 2010;107:659–666. doi: 10.1161/circresaha.110.220038 20616315 PMC2933281

[67] Wu X, Zhang T, Bossuyt J, Li X, McKinsey TA, Dedman JR, Olson EN, Chen J, Brown JH, Bers DM. Local InsP3-dependent perinuclear Ca2+ signaling in cardiac myocyte excitation-transcription coupling. J Clin Invest. 2006;116:675–682. doi: 10.1172/JCI27374 16511602 PMC1386110

[68] Braz JC, Gregory K, Pathak A, Zhao W, Sahin B, Klevitsky R, Kimball TF, Lorenz JN, Nairn AC, Liggett SB, PKC-alpha regulates cardiac contractility and propensity toward heart failure. Nat Med. 2004;10:248–254. doi: 10.1038/nm1000 14966518

[69] Kozasa T, Jiang X, Hart MJ, Sternweis PM, Singer WD, Gilman AG, Bollag G, Sternweis PC. p115 RhoGEF, a GTPase activating protein for Galpha12 and Galpha13. Science. 1998;280:2109–2111. doi: doi:10.1126/science.280.5372.2109 9641915

[70] Hart MJ, Jiang X, Kozasa T, Roscoe W, Singer WD, Gilman AG, Sternweis PC, Bollag G. Direct stimulation of the guanine nucleotide exchange activity of p115 RhoGEF by Galpha13. Science. 1998;280:2112–2114. doi: 10.1126/science.280.5372.2112 9641916

[71] Gohla A, Schultz G, Offermanns S. Role for G(12)/G(13) in agonist-induced vascular smooth muscle cell contraction. Circ Res. 2000;87:221–227. doi: 10.1161/01.res.87.3.221 10926873

[72] Wirth A, Benyo Z, Lukasova M, Leutgeb B, Wettschureck N, Gorbey S, Orsy P, Horvath B, Maser-Gluth C, Greiner E, G12-G13-LARG-mediated signaling in vascular smooth muscle is required for salt-induced hypertension. Nat Med. 2008;14:64–68. doi: 10.1038/nm1666 18084302

[73] Suzuki N, Hajicek N, Kozasa T. Regulation and physiological functions of G12/13-mediated signaling pathways. Neurosignals. 2009;17:55–70. doi: 10.1159/000186690 19212140 PMC2836950

[74] Ford CE, Skiba NP, Bae H, Daaka Y, Reuveny E, Shekter LR, Rosal R, Weng G, Yang CS, Iyengar R, Molecular basis for interactions of G protein betagamma subunits with effectors. Science. 1998;280:1271–1274. doi: 10.1126/science.280.5367.1271 9596582

[75] Masuho I, Skamangas NK, Muntean BS, Martemyanov KA. Diversity of the Gbetagamma complexes defines spatial and temporal bias of GPCR signaling. Cell Syst. 2021;12:324–337 e325. doi: 10.1016/j.cels.2021.02.001 33667409 PMC8068604

[76] Rockman HA, Koch WJ, Lefkowitz RJ. Seven-transmembrane-spanning receptors and heart function. Nature. 2002;415:206–212. doi: 10.1038/415206a 11805844

[77] Zhong H, Neubig RR. Regulator of G protein signaling proteins: novel multifunctional drug targets. J Pharmacol Exp Ther. 2001;297:837–845. 11356902

[78] Pitcher JA, Freedman NJ, Lefkowitz RJ. G PROTEIN–COUPLED RECEPTOR KINASES. Annual Review of Biochemistry. 1998;67:653–692. doi: 10.1146/annurev.biochem.67.1.653 9759500

[79] Rajagopal S, Shenoy SK. GPCR desensitization: Acute and prolonged phases. Cell Signal. 2018;41:9–16. doi: 10.1016/j.cellsig.2017.01.024 28137506 PMC5533627

[80] Benovic JL, Pike LJ, Cerione RA, Staniszewski C, Yoshimasa T, Codina J, Caron MG, Lefkowitz RJ. Phosphorylation of the mammalian beta-adrenergic receptor by cyclic AMP-dependent protein kinase. Regulation of the rate of receptor phosphorylation and dephosphorylation by agonist occupancy and effects on coupling of the receptor to the stimulatory guanine nucleotide regulatory protein. J Biol Chem. 1985;260:7094–7101. 2987243

[81] Daaka Y, Luttrell LM, Lefkowitz RJ. Switching of the coupling of the beta2-adrenergic receptor to different G proteins by protein kinase A. Nature. 1997;390:88–91. doi: 10.1038/36362 9363896

[82] Oduori OS, Murao N, Shimomura K, Takahashi H, Zhang Q, Dou H, Sakai S, Minami K, Chanclon B, Guida C, Gs/Gq signaling switch in beta cells defines incretin effectiveness in diabetes. J Clin Invest. 2020;130:6639–6655. doi: 10.1172/JCI140046 33196462 PMC7685756

[83] Komolov KE, Benovic JL. G protein-coupled receptor kinases: Past, present and future. Cell Signal. 2018;41:17–24. doi: 10.1016/j.cellsig.2017.07.004 28711719 PMC5722692

[84] Mushegian A, Gurevich VV, Gurevich EV. The origin and evolution of G protein-coupled receptor kinases. PLoS One. 2012;7:e33806. doi: 10.1371/journal.pone.0033806 22442725 PMC3307776

[85] Touhara K, Inglese J, Pitcher JA, Shaw G, Lefkowitz RJ. Binding of G protein beta gamma-subunits to pleckstrin homology domains. J Biol Chem. 1994;269:10217–10220. 8144601

[86] Butcher AJ, Prihandoko R, Kong KC, McWilliams P, Edwards JM, Bottrill A, Mistry S, Tobin AB. Differential G-protein-coupled receptor phosphorylation provides evidence for a signaling bar code. J Biol Chem. 2011;286:11506–11518. doi: 10.1074/jbc.M110.154526 21177246 PMC3064205

[87] Nobles KN, Xiao K, Ahn S, Shukla AK, Lam CM, Rajagopal S, Strachan RT, Huang TY, Bressler EA, Hara MR, Distinct phosphorylation sites on the beta(2)-adrenergic receptor establish a barcode that encodes differential functions of beta-arrestin. Sci Signal. 2011;4:ra51. doi: 10.1126/scisignal.2001707 21868357 PMC3415961

[88] Yang Z, Yang F, Zhang D, Liu Z, Lin A, Liu C, Xiao P, Yu X, Sun JP. Phosphorylation of G Protein-Coupled Receptors: From the Barcode Hypothesis to the Flute Model. Mol Pharmacol. 2017;92:201–210. doi: 10.1124/mol.116.107839 28246190

[89] Cant SH, Pitcher JA. G protein-coupled receptor kinase 2-mediated phosphorylation of ezrin is required for G protein-coupled receptor-dependent reorganization of the actin cytoskeleton. Mol Biol Cell. 2005;16:3088–3099. doi: 10.1091/mbc.e04-10-0877 15843435 PMC1165394

[90] Naga Prasad SV, Laporte SA, Chamberlain D, Caron MG, Barak L, Rockman HA. Phosphoinositide 3-kinase regulates beta2-adrenergic receptor endocytosis by AP-2 recruitment to the receptor/beta-arrestin complex. J Cell Biol. 2002;158:563–575. doi: 10.1083/jcb.200202113 12163475 PMC2173831

[91] Penela P, Murga C, Ribas C, Lafarga V, Mayor F Jr. The complex G protein-coupled receptor kinase 2 (GRK2) interactome unveils new physiopathological targets. Br J Pharmacol. 2010;160:821–832. doi: 10.1111/j.1476-5381.2010.00727.x 20590581 PMC2935989

[92] Kurosawa A, Guidotti A, Costa E. Nuclear translocation of cyclic AMP-dependent protein kinase subunits during the transsynaptic activation of gene expression in rat adrenal medulla. Mol Pharmacol. 1979;15:115–130. 218090

[93] Schwartz JP, Costa E. Protein kinase translocation following beta-adrenergic receptor activation in C6 glioma cells. J Biol Chem. 1980;255:2943–2948. 6244301

[94] Martini JS, Raake P, Vinge LE, DeGeorge BR Jr., Chuprun JK, Harris DM, Gao E, Eckhart AD, Pitcher JA, Koch WJ. Uncovering G protein-coupled receptor kinase-5 as a histone deacetylase kinase in the nucleus of cardiomyocytes. Proc Natl Acad Sci U S A. 2008;105:12457–12462. doi: 10.1073/pnas.0803153105 18711143 PMC2527933

[95] Pfleger J, Gresham K, Koch WJ. G protein-coupled receptor kinases as therapeutic targets in the heart. Nat Rev Cardiol. 2019;16:612–622. doi: 10.1038/s41569-019-0220-3 31186538

[96] Lefkowitz RJ, Rajagopal K, Whalen EJ. New roles for beta-arrestins in cell signaling: not just for seven-transmembrane receptors. Mol Cell. 2006;24:643–652. doi: 10.1016/j.molcel.2006.11.007 17157248

[97] Wang P, Wu Y, Ge X, Ma L, Pei G. Subcellular localization of beta-arrestins is determined by their intact N domain and the nuclear export signal at the C terminus. J Biol Chem. 2003;278:11648–11653. doi: 10.1074/jbc.M208109200 12538596

[98] Shukla AK, Westfield GH, Xiao K, Reis RI, Huang LY, Tripathi-Shukla P, Qian J, Li S, Blanc A, Oleskie AN, Visualization of arrestin recruitment by a G-protein-coupled receptor. Nature. 2014;512:218–222. doi: 10.1038/nature13430 25043026 PMC4134437

[99] Maharana J, Sano FK, Sarma P, Yadav MK, Duan L, Stepniewski TM, Chaturvedi M, Ranjan A, Singh V, Saha S, Molecular insights into atypical modes of β-arrestin interaction with seven transmembrane receptors. Science. 2024;383:101–108. doi: 10.1126/science.adj3347 38175886 PMC7615931

[100] Xiao K, McClatchy DB, Shukla AK, Zhao Y, Chen M, Shenoy SK, Yates JR, Lefkowitz RJ. Functional specialization of β-arrestin interactions revealed by proteomic analysis. Proc Natl Acad Sci U S A. 2007;104:12011–12016. doi: doi:10.1073/pnas.0704849104 17620599 PMC1913545

[101] Ahn S, Shenoy SK, Luttrell LM, Lefkowitz RJ. SnapShot: β-arrestin functions. Cell. 2020;182:1362–1362.e1361. doi: 10.1016/j.cell.2020.07.034 32888497

[102] Oakley RH, Laporte SA, Holt JA, Barak LS, Caron MG. Association of beta-arrestin with G protein-coupled receptors during clathrin-mediated endocytosis dictates the profile of receptor resensitization. J Biol Chem. 1999;274:32248–32257. doi: 10.1074/jbc.274.45.32248 10542263

[103] Laporte SA, Oakley RH, Holt JA, Barak LS, Caron MG. The interaction of beta-arrestin with the AP-2 adaptor is required for the clustering of beta 2-adrenergic receptor into clathrin-coated pits. J Biol Chem. 2000;275:23120–23126. doi: 10.1074/jbc.M002581200 10770944

[104] Tohgo A, Pierce KL, Choy EW, Lefkowitz RJ, Luttrell LM. Beta-Arrestin scaffolding of the ERK cascade enhances cytosolic ERK activity but inhibits ERK-mediated transcription following angiotensin AT1a receptor stimulation. J Biol Chem. 2002;277:9429–9436. doi: 10.1074/jbc.M106457200 11777902

[105] Beaulieu JM, Sotnikova TD, Marion S, Lefkowitz RJ, Gainetdinov RR, Caron MG. An Akt/beta-arrestin 2/PP2A signaling complex mediates dopaminergic neurotransmission and behavior. Cell. 2005;122:261–273. doi: 10.1016/j.cell.2005.05.012 16051150

[106] Shenoy SK, Drake MT, Nelson CD, Houtz DA, Xiao K, Madabushi S, Reiter E, Premont RT, Lichtarge O, Lefkowitz RJ. Beta-arrestin-dependent, G protein-independent ERK1/2 activation by the beta2 adrenergic receptor. J Biol Chem. 2006;281:1261–1273. doi: 10.1074/jbc.M506576200 16280323

[107] Ferrero KM, Koch WJ. GRK2 in cardiovascular disease and its potential as a therapeutic target. J Mol Cell Cardiol. 2022;172:14–23. doi: 10.1016/j.yjmcc.2022.07.008 35878706 PMC12186197

[108] Shenoy SK, Lefkowitz RJ. Seven-transmembrane receptor signaling through beta-arrestin. Sci STKE. 2005;2005:cm10. doi: 10.1126/stke.2005/308/cm10 16267056

[109] Ahn S, Shenoy SK, Wei H, Lefkowitz RJ. Differential kinetic and spatial patterns of beta-arrestin and G protein-mediated ERK activation by the angiotensin II receptor. J Biol Chem. 2004;279:35518–35525. doi: 10.1074/jbc.M405878200 15205453

[110] Noma T, Lemaire A, Naga Prasad SV, Barki-Harrington L, Tilley DG, Chen J, Le Corvoisier P, Violin JD, Wei H, Lefkowitz RJ, Beta-arrestin-mediated beta1-adrenergic receptor transactivation of the EGFR confers cardioprotection. J Clin Invest. 2007;117:2445–2458. doi: 10.1172/jci31901 17786238 PMC1952636

[111] Tilley DG, Kim IM, Patel PA, Violin JD, Rockman HA. Beta-Arrestin mediates beta1-adrenergic receptor-epidermal growth factor receptor interaction and downstream signaling. J Biol Chem. 2009;284:20375–20386. doi: 10.1074/jbc.M109.005793 19509284 PMC2740462

[112] Wei H, Ahn S, Shenoy SK, Karnik SS, Hunyady L, Luttrell LM, Lefkowitz RJ. Independent beta-arrestin 2 and G protein-mediated pathways for angiotensin II activation of extracellular signal-regulated kinases 1 and 2. Proc Natl Acad Sci U S A. 2003;100:10782–10787. doi: 10.1073/pnas.1834556100 12949261 PMC196880

[113] Ahn S, Wei H, Garrison TR, Lefkowitz RJ. Reciprocal regulation of angiotensin receptor-activated extracellular signal-regulated kinases by beta-arrestins 1 and 2. J Biol Chem. 2004;279:7807–7811. doi: 10.1074/jbc.C300443200 C300443200 [pii] 14711824

[114] Feinstein TN, Yui N, Webber MJ, Wehbi VL, Stevenson HP, King JD Jr., Hallows KR, Brown D, Bouley R, Vilardaga JP. Noncanonical control of vasopressin receptor type 2 signaling by retromer and arrestin. J Biol Chem. 2013;288:27849–27860. doi: 10.1074/jbc.M112.445098 23935101 PMC3784700

[115] Thomsen ARB, Plouffe B, Cahill TJ 3rd, Shukla, Tarrasch JT, Dosey AM, Kahsai AW, Strachan RT, Pani B, Mahoney JP, GPCR-G Protein-beta-Arrestin Super-Complex Mediates Sustained G Protein Signaling. Cell. 2016;166:907–919. doi: 10.1016/j.cell.2016.07.004 27499021 PMC5418658

[116] Ritter SL, Hall RA. Fine-tuning of GPCR activity by receptor-interacting proteins. Nature reviews Molecular cell biology. 2009;10:819–830. doi: 10.1038/nrm2803 19935667 PMC2825052

[117] McLatchie LM, Fraser NJ, Main MJ, Wise A, Brown J, Thompson N, Solari R, Lee MG, Foord SM. RAMPs regulate the transport and ligand specificity of the calcitonin-receptor-like receptor. Nature. 1998;393:333–339. doi: 10.1038/30666 9620797

[118] Russell FA, King R, Smillie SJ, Kodji X, Brain SD. Calcitonin gene-related peptide: physiology and pathophysiology. Physiol Rev. 2014;94:1099–1142. doi: 10.1152/physrev.00034.2013 25287861 PMC4187032

[119] Lorenzen E, Dodig-Crnkovic T, Kotliar IB, Pin E, Ceraudo E, Vaughan RD, Uhlen M, Huber T, Schwenk JM, Sakmar TP. Multiplexed analysis of the secretin-like GPCR-RAMP interactome. Sci Adv. 2019;5:eaaw2778. doi: 10.1126/sciadv.aaw2778 31555726 PMC6750928

[120] Mackie DI, Nielsen NR, Harris M, Singh S, Davis RB, Dy D, Ladds G, Caron KM. RAMP3 determines rapid recycling of atypical chemokine receptor-3 for guided angiogenesis. Proc Natl Acad Sci U S A. 2019;116:24093–24099. doi: 10.1073/pnas.1905561116 31712427 PMC6883789

[121] Smith JS, Lefkowitz RJ, Rajagopal S. Biased signalling: from simple switches to allosteric microprocessors. Nat Rev Drug Discov. 2018;17:243–260. doi: 10.1038/nrd.2017.229 29302067 PMC5936084

[122] Kolb P, Kenakin T, Alexander SPH, Bermudez M, Bohn LM, Breinholt CS, Bouvier M, Hill SJ, Kostenis E, Martemyanov KA, Community guidelines for GPCR ligand bias: IUPHAR review 32. Br J Pharmacol. 2022;179:3651–3674. doi: 10.1111/bph.15811 35106752 PMC7612872

[123] Wingler LM, Elgeti M, Hilger D, Latorraca NR, Lerch MT, Staus DP, Dror RO, Kobilka BK, Hubbell WL, Lefkowitz RJ. Angiotensin analogs with divergent bias stabilize distinct receptor conformations. Cell. 2019;176:468–478 e411. doi: 10.1016/j.cell.2018.12.005 30639099 PMC6475118

[124] Yang LK, Hou ZS, Tao YX. Biased signaling in naturally occurring mutations of G protein-coupled receptors associated with diverse human diseases. Biochim Biophys Acta Mol Basis Dis. 2021;1867:165973. doi: 10.1016/j.bbadis.2020.165973 32949766 PMC7722056

[125] Lymperopoulos A, Rengo G, Koch WJ. GRK2 inhibition in heart failure: something old, something new. Curr Pharm Des. 2012;18:186–191. doi: 10.2174/138161212799040510 22229578

[126] Wang J, Gareri C, Rockman HA. G-Protein-Coupled Receptors in Heart Disease. Circ Res. 2018;123:716–735. doi: 10.1161/CIRCRESAHA.118.311403 30355236 PMC6205195

[127] Eiger DS, Hicks C, Gardner J, Pham U, Rajagopal S. Location bias: A “Hidden Variable” in GPCR pharmacology. Bioessays. 2023;45:e2300123. doi: 10.1002/bies.202300123 37625014 PMC11900906

[128] Monasky MM, Taglieri DM, Henze M, Warren CM, Utter MS, Soergel DG, Violin JD, Solaro RJ. The β-arrestin-biased ligand TRV120023 inhibits angiotensin II-induced cardiac hypertrophy while preserving enhanced myofilament response to calcium. Am J Physiol Heart Circ Physiol. 2013;305:H856–866. doi: 10.1152/ajpheart.00327.2013 23873795 PMC3761345

[129] Boivin B, Lavoie C, Vaniotis G, Baragli A, Villeneuve L-R, Ethier N, Trieu P, Allen BG, Hébert TE. Functional β-adrenergic receptor signalling on nuclear membranes in adult rat and mouse ventricular cardiomyocytes. Cardiovascular Research. 2006;71:69–78. doi: 10.1016/j.cardiores.2006.03.015 16631628

[130] Huang Y, Wright CD, Merkwan CL, Baye NL, Liang Q, Simpson PC, O’Connell TD. An alpha1A-adrenergic-extracellular signal-regulated kinase survival signaling pathway in cardiac myocytes. Circulation. 2007;115:763–772. doi: 10.1161/CIRCULATIONAHA.106.664862 17283256

[131] Tadevosyan A, Vaniotis G, Allen BG, Hebert TE, Nattel S. G protein-coupled receptor signalling in the cardiac nuclear membrane: evidence and possible roles in physiological and pathophysiological function. J Physiol. 2012;590:1313–1330. doi: 10.1113/jphysiol.2011.222794 22183719 PMC3382322

[132] Hakak Y, Shrestha D, Goegel MC, Behan DP, Chalmers DT. Global analysis of G-protein-coupled receptor signaling in human tissues. FEBS Letters. 2003;550:11–17. doi: 10.1016/s0014-5793(03)00762-2 12935878

[133] Mann DL, Bristow MR. Mechanisms and models in heart failure: the biomechanical model and beyond. Circulation. 2005;111:2837–2849. doi: 10.1161/circulationaha.104.500546 15927992

[134] Chen ZM, Pan HC, Chen YP, Peto R, Collins R, Jiang LX, Xie JX, Liu LS. Early intravenous then oral metoprolol in 45,852 patients with acute myocardial infarction: randomised placebo-controlled trial. Lancet. 2005;366:1622–1632. doi: 10.1016/s0140-6736(05)67661-1 16271643

[135] Foody JM, Farrell MH, Krumholz HM. Beta-Blocker therapy in heart failure: scientific review. JAMA. 2002;287:883–889. doi: 10.1001/jama.287.7.883 11851582

[136] Wisler JW, DeWire SM, Whalen EJ, Violin JD, Drake MT, Ahn S, Shenoy SK, Lefkowitz RJ. A unique mechanism of beta-blocker action: carvedilol stimulates beta-arrestin signaling. Proc Natl Acad Sci U S A. 2007;104:16657–16662. doi: 10.1073/pnas.0707936104 17925438 PMC2034221

[137] Kim IM, Tilley DG, Chen J, Salazar NC, Whalen EJ, Violin JD, Rockman HA. Beta-blockers alprenolol and carvedilol stimulate beta-arrestin-mediated EGFR transactivation. Proc Natl Acad Sci U S A. 2008;105:14555–14560. doi: 10.1073/pnas.0804745105 18787115 PMC2567217

[138] Kim IM, Wang Y, Park KM, Tang Y, Teoh JP, Vinson J, Traynham CJ, Pironti G, Mao L, Su H, β-arrestin1-biased β1-adrenergic receptor signaling regulates microRNA processing. Circ Res. 2014;114:833–844. doi: 10.1161/circresaha.114.302766 24334028 PMC3955054

[139] Wang J, Hanada K, Staus DP, Makara MA, Dahal GR, Chen Q, Ahles A, Engelhardt S, Rockman HA. Gα(i) is required for carvedilol-induced β(1) adrenergic receptor β-arrestin biased signaling. Nat Commun. 2017;8:1706. doi: 10.1038/s41467-017-01855-z 29167435 PMC5700200

[140] Wang J, Pani B, Gokhan I, Xiong X, Kahsai AW, Jiang H, Ahn S, Lefkowitz RJ, Rockman HA. β-arrestin-biased allosteric modulator potentiates Carvedilol-stimulated β adrenergic receptor cardioprotection. Mol Pharmacol. 2021;100:568–579. doi: 10.1124/molpharm.121.000359 34561298 PMC8626783

[141] Benkel T, Zimmermann M, Zeiner J, Bravo S, Merten N, Lim VJY, Matthees ESF, Drube J, Miess-Tanneberg E, Malan D, How Carvedilol activates beta(2)-adrenoceptors. Nat Commun. 2022;13:7109. doi: 10.1038/s41467-022-34765-w 36402762 PMC9675828

[142] Yoshikawa T, Port JD, Asano K, Chidiak P, Bouvier M, Dutcher D, Roden RL, Minobe W, Tremmel KD, Bristow MR. Cardiac adrenergic receptor effects of carvedilol. Eur Heart J. 1996;17 Suppl B:8–16. doi: 10.1093/eurheartj/17.suppl_b.8 8733065

[143] Feuerstein GZ, Bril A, Ruffolo RR Jr. Protective effects of carvedilol in the myocardium. Am J Cardiol. 1997;80:41l–45l. doi: 10.1016/s0002-9149(97)00847-3 9412541

[144] Kim J, Grotegut CA, Wisler JW, Mao L, Rosenberg PB, Rockman HA, Lefkowitz RJ. The β-arrestin-biased β-adrenergic receptor blocker carvedilol enhances skeletal muscle contractility. Proc Natl Acad Sci U S A. 2020;117:12435–12443. doi: 10.1073/pnas.1920310117 32414934 PMC7275696

[145] Salazar NC, Chen J, Rockman HA. Cardiac GPCRs: GPCR signaling in healthy and failing hearts. Biochim Biophys Acta. 2007;1768:1006–1018. doi: 10.1016/j.bbamem.2007.02.010 17376402 PMC1892229

[146] Haywood GA, Gullestad L, Katsuya T, Hutchinson HG, Pratt RE, Horiuchi M, Fowler MB. AT1 and AT2 angiotensin receptor gene expression in human heart failure. Circulation. 1997;95:1201–1206. doi: 10.1161/01.cir.95.5.1201 9054850

[147] Luttrell LM, Roudabush FL, Choy EW, Miller WE, Field ME, Pierce KL, Lefkowitz RJ. Activation and targeting of extracellular signal-regulated kinases by beta-arrestin scaffolds. Proc Natl Acad Sci U S A. 2001;98:2449–2454. doi: 10.1073/pnas.041604898 11226259 PMC30158

[148] Fessart D, Simaan M, Laporte SA. c-Src regulates clathrin adapter protein 2 interaction with beta-arrestin and the angiotensin II type 1 receptor during clathrin- mediated internalization. Mol Endocrinol. 2005;19:491–503. doi: 10.1210/me.2004-0246 15498833

[149] Pfeiffer CT, Wang J, Paulo JA, Jiang X, Gygi SP, Rockman HA. Mapping angiotensin II type 1 receptor-biased signaling using proximity labeling and proteomics identifies diverse actions of biased agonists. J Proteome Res. 2021;20:3256–3267. doi: 10.1021/acs.jproteome.1c00080 33950683 PMC8218870

[150] Xiao K, Sun J, Kim J, Rajagopal S, Zhai B, Villen J, Haas W, Kovacs JJ, Shukla AK, Hara MR, Global phosphorylation analysis of beta-arrestin-mediated signaling downstream of a seven transmembrane receptor (7TMR). Proc Natl Acad Sci U S A. 2010;107:15299–15304. doi: 10.1073/pnas.1008461107 20686112 PMC2930550

[151] Rakesh K, Yoo B, Kim IM, Salazar N, Kim KS, Rockman HA. beta-Arrestin-biased agonism of the angiotensin receptor induced by mechanical stress. Sci Signal. 2010;3:ra46. doi: 10.1126/scisignal.2000769 20530803 PMC2981501

[152] Tang W, Strachan RT, Lefkowitz RJ, Rockman HA. Allosteric modulation of β-arrestin-biased angiotensin II type 1 receptor signaling by membrane stretch. J Biol Chem. 2014;289:28271–28283. doi: 10.1074/jbc.M114.585067 25170081 PMC4192482

[153] Gekle M, Dubourg V, Schwerdt G, Benndorf RA, Schreier B. The role of EGFR in vascular AT1R signaling: From cellular mechanisms to systemic relevance. Biochem Pharmacol. 2023;217:115837. doi: 10.1016/j.bcp.2023.115837 37777161

[154] Yancy CW, Jessup M, Bozkurt B, Butler J, Casey DE Jr., Colvin MM, Drazner MH, Filippatos GS, Fonarow GC, Givertz MM, 2017 ACC/AHA/HFSA Focused Update of the 2013 ACCF/AHA Guideline for the Management of Heart Failure: A Report of the American College of Cardiology/American Heart Association Task Force on Clinical Practice Guidelines and the Heart Failure Society of America. J Am Coll Cardiol. 2017;70:776–803. doi: 10.1016/j.jacc.2017.04.025 28461007

[155] Whelton PK, Carey RM, Aronow WS, Casey DE Jr., Collins KJ, Dennison Himmelfarb C, DePalma SM, Gidding S, Jamerson KA, Jones, 2017 ACC/AHA/AAPA/ABC/ACPM/AGS/APhA/ASH/ASPC/NMA/PCNA Guideline for the Prevention, Detection, Evaluation, and Management of High Blood Pressure in Adults: A Report of the American College of Cardiology/American Heart Association Task Force on Clinical Practice Guidelines. Hypertension. 2018;71:e13–e115. doi: 10.1161/hyp.0000000000000065 29133356

[156] Chen R, Suchard MA, Krumholz HM, Schuemie MJ, Shea S, Duke J, Pratt N, Reich CG, Madigan D, You SC, Comparative first-line effectiveness and safety of ACE (Angiotensin-Converting Enzyme) inhibitors and angiotensin receptor blockers: a multinational cohort study. Hypertension. 2021;78:591–603. doi: 10.1161/HYPERTENSIONAHA.120.16667 34304580 PMC8363588

[157] Effects of enalapril on mortality in severe congestive heart failure. Results of the Cooperative North Scandinavian Enalapril Survival Study (CONSENSUS). N Engl J Med. 1987;316:1429–1435. doi: 10.1056/nejm198706043162301 2883575

[158] Pitt B, Segal R, Martinez FA, Meurers G, Cowley AJ, Thomas I, Deedwania PC, Ney DE, Snavely DB, Chang PI. Randomised trial of losartan versus captopril in patients over 65 with heart failure (Evaluation of Losartan in the Elderly Study, ELITE). Lancet. 1997;349:747–752. doi: 10.1016/s0140-6736(97)01187-2 9074572

[159] McMurray JJ, Packer M, Desai AS, Gong J, Lefkowitz MP, Rizkala AR, Rouleau JL, Shi VC, Solomon SD, Swedberg K, Angiotensin-neprilysin inhibition versus enalapril in heart failure. N Engl J Med. 2014;371:993–1004. doi: 10.1056/NEJMoa1409077 25176015

[160] Rajagopal K, Whalen EJ, Violin JD, Stiber JA, Rosenberg PB, Premont RT, Coffman TM, Rockman HA, Lefkowitz RJ. β-Arrestin2-mediated inotropic effects of the angiotensin II type 1A receptor in isolated cardiac myocytes. Proc Natl Acad Sci U S A. 2006;103:16284–16289. doi: doi:10.1073/pnas.0607583103 17060617 PMC1637574

[161] Saulière A, Bellot M, Paris H, Denis C, Finana F, Hansen JT, Altié MF, Seguelas MH, Pathak A, Hansen JL, Deciphering biased-agonism complexity reveals a new active AT1 receptor entity. Nat Chem Biol. 2012;8:622–630. doi: 10.1038/nchembio.961 22634635

[162] Akhter SA, Luttrell LM, Rockman HA, Iaccarino G, Lefkowitz RJ, Koch WJ. Targeting the receptor-Gq interface to inhibit in vivo pressure overload myocardial hypertrophy. Science. 1998;280:574–577. doi: doi:10.1126/science.280.5363.574 9554846

[163] Violin JD, DeWire SM, Yamashita D, Rominger DH, Nguyen L, Schiller K, Whalen EJ, Gowen M, Lark MW. Selectively engaging β-arrestins at the angiotensin II type 1 receptor reduces blood pressure and increases cardiac performance. J Pharmacol Exp Ther. 2010;335:572–579. doi: 10.1124/jpet.110.173005 20801892

[164] Boerrigter G, Soergel DG, Violin JD, Lark MW, Burnett JC, Jr. TRV120027, a novel β-arrestin biased ligand at the angiotensin II type I receptor, unloads the heart and maintains renal function when added to furosemide in experimental heart failure. Circ Heart Fail. 2012;5:627–634. doi: 10.1161/circheartfailure.112.969220 22891045

[165] Kim KS, Abraham D, Williams B, Violin JD, Mao L, Rockman HA. β-Arrestin-biased AT1R stimulation promotes cell survival during acute cardiac injury. Am J Physiol Heart Circ Physiol. 2012;303:H1001–1010. doi: 10.1152/ajpheart.00475.2012 22886417 PMC3469643

[166] Abraham DM, Davis RT 3rd, Warren CM, Mao L, Wolska BM, Solaro RJ, Rockman HA. β-Arrestin mediates the Frank-Starling mechanism of cardiac contractility. Proc Natl Acad Sci U S A. 2016;113:14426–14431. doi: 10.1073/pnas.1609308113 27911784 PMC5167158

[167] Pang PS, Butler J, Collins SP, Cotter G, Davison BA, Ezekowitz JA, Filippatos G, Levy PD, Metra M, Ponikowski P, Biased ligand of the angiotensin II type 1 receptor in patients with acute heart failure: a randomized, double-blind, placebo-controlled, phase IIB, dose ranging trial (BLAST-AHF). Eur Heart J. 2017;38:2364–2373. doi: 10.1093/eurheartj/ehx196 28459958 PMC5837312

[168] Cotter G, Davison BA, Butler J, Collins SP, Ezekowitz JA, Felker GM, Filippatos G, Levy PD, Metra M, Ponikowski P, Relationship between baseline systolic blood pressure and long-term outcomes in acute heart failure patients treated with TRV027: an exploratory subgroup analysis of BLAST-AHF. Clin Res Cardiol. 2018;107:170–181. doi: 10.1007/s00392-017-1168-0 28986703

[169] Sugihara S, Burnett JC Jr. BLAST-AHF: insights into biased AT1 ligands and heart failure. Beginning of the end or end of the beginning? Eur Heart J. 2017;38:2374–2376. doi: 10.1093/eurheartj/ehx276 28575396 PMC5837525

[170] Campbell JE, Drucker DJ. Pharmacology, physiology, and mechanisms of incretin hormone action. Cell Metab. 2013;17:819–837. doi: 10.1016/j.cmet.2013.04.008 23684623

[171] Ussher JR, Drucker DJ. Glucagon-like peptide 1 receptor agonists: cardiovascular benefits and mechanisms of action. Nat Rev Cardiol. 2023;20:463–474. doi: 10.1038/s41569-023-00849-3 36977782

[172] Sattar N, Lee MMY, Kristensen SL, Branch KRH, Del Prato S, Khurmi NS, Lam CSP, Lopes RD, McMurray JJV, Pratley RE, Cardiovascular, mortality, and kidney outcomes with GLP-1 receptor agonists in patients with type 2 diabetes: a systematic review and meta-analysis of randomised trials. Lancet Diabetes Endocrinol. 2021;9:653–662. doi: 10.1016/s2213-8587(21)00203-5 34425083

[173] Lincoff AM, Brown-Frandsen K, Colhoun HM, Deanfield J, Emerson SS, Esbjerg S, Hardt-Lindberg S, Hovingh GK, Kahn SE, Kushner RF, Semaglutide and cardiovascular outcomes in obesity without diabetes. N Engl J Med. 2023;389:2221–2232. doi: 10.1056/NEJMoa2307563 37952131

[174] Kosiborod MN, Abildstrøm SZ, Borlaug BA, Butler J, Rasmussen S, Davies M, Hovingh GK, Kitzman DW, Lindegaard ML, Møller DV, Semaglutide in patients with heart failure with preserved ejection fraction and obesity. N Engl J Med. 2023;389:1069–1084. doi: 10.1056/NEJMoa2306963 37622681

[175] Couzin-Frankel J Obesity meets its match. Science. 2023;382:1226–1227. doi: 10.1126/science.adn4691 38096291

[176] Powell-Wiley TM, Poirier P, Burke LE, Després JP, Gordon-Larsen P, Lavie CJ, Lear SA, Ndumele CE, Neeland IJ, Sanders P, Obesity and cardiovascular disease: a scientific statement from the American Heart Association. Circulation. 2021;143:e984–e1010. doi: 10.1161/cir.0000000000000973 33882682 PMC8493650

[177] Silverman DN, Litwin SE. Pharmacologic Weight Loss for Heart Failure With Preserved Ejection Fraction: Getting to the Core of the Problem. Circ Heart Fail. 2021;14:e008554. doi: 10.1161/circheartfailure.121.008554 34517721

[178] Cameron-Vendrig A, Reheman A, Siraj MA, Xu XR, Wang Y, Lei X, Afroze T, Shikatani E, El-Mounayri O, Noyan H, Glucagon-Like Peptide 1 Receptor Activation Attenuates Platelet Aggregation and Thrombosis. Diabetes. 2016;65:1714–1723. doi: 10.2337/db15-1141 26936963

[179] Cahill KN, Amin T, Boutaud O, Printz R, Newcomb DC, Foer D, Hodson DJ, Broichhagen J, Beckman JA, Yu C, Glucagon-Like Peptide-1 Receptor Regulates Thromboxane-Induced Human Platelet Activation. JACC Basic Transl Sci. 2022;7:713–715. doi: 10.1016/j.jacbts.2022.04.004 35958685 PMC9357570

[180] Clarke SJ, Giblett JP, Yang LL, Hubsch A, Zhao T, Aetesam-Ur-Rahman M, West NEJ, O’Sullivan M, Figg N, Bennett M, GLP-1 Is a Coronary Artery Vasodilator in Humans. J Am Heart Assoc. 2018;7:e010321. doi: 10.1161/jaha.118.010321 30571482 PMC6404441

[181] Helmstädter J, Frenis K, Filippou K, Grill A, Dib M, Kalinovic S, Pawelke F, Kus K, Kröller-Schön S, Oelze M, Endothelial GLP-1 (Glucagon-Like Peptide-1) Receptor Mediates Cardiovascular Protection by Liraglutide In Mice With Experimental Arterial Hypertension. Arterioscler Thromb Vasc Biol. 2020;40:145–158. doi: 10.1161/atv.0000615456.97862.30 31747801 PMC6946108

[182] Saxena AR, Gorman DN, Esquejo RM, Bergman A, Chidsey K, Buckeridge C, Griffith DA, Kim AM. Danuglipron (PF-06882961) in type 2 diabetes: a randomized, placebo-controlled, multiple ascending-dose phase 1 trial. Nature Medicine. 2021;27:1079–1087. doi: 10.1038/s41591-021-01391-w 34127852

[183] Griffith DA, Edmonds DJ, Fortin JP, Kalgutkar AS, Kuzmiski JB, Loria PM, Saxena AR, Bagley SW, Buckeridge C, Curto JM, A small-molecule oral agonist of the human glucagon-like peptide-1 receptor. J Med Chem. 2022;65:8208–8226. doi: 10.1021/acs.jmedchem.1c01856 35647711 PMC9234956

[184] Coskun T, Urva S, Roell WC, Qu H, Loghin C, Moyers JS, O’Farrell LS, Briere DA, Sloop KW, Thomas MK, LY3437943, a novel triple glucagon, GIP, and GLP-1 receptor agonist for glycemic control and weight loss: From discovery to clinical proof of concept. Cell Metab. 2022;34:1234–1247.e1239. doi: 10.1016/j.cmet.2022.07.013 35985340

[185] Jastreboff AM, Kaplan LM, Frías JP, Wu Q, Du Y, Gurbuz S, Coskun T, Haupt A, Milicevic Z, Hartman ML. Triple-hormone-receptor agonist retatrutide for obesity - a phase 2 trial. N Engl J Med. 2023;389:514–526. doi: 10.1056/NEJMoa2301972 37366315

[186] Wright SC, Motso A, Koutsilieri S, Beusch CM, Sabatier P, Berghella A, Blondel-Tepaz É, Mangenot K, Pittarokoilis I, Sismanoglou DC, GLP-1R signaling neighborhoods associate with the susceptibility to adverse drug reactions of incretin mimetics. Nat Commun. 2023;14:6243. doi: 10.1038/s41467-023-41893-4 37813859 PMC10562414

[187] Willard FS, Douros JD, Gabe MB, Showalter AD, Wainscott DB, Suter TM, Capozzi ME, van der Velden WJ, Stutsman C, Cardona GR, Tirzepatide is an imbalanced and biased dual GIP and GLP-1 receptor agonist. JCI Insight. 2020;5:e140532. doi: 10.1172/jci.insight.140532 32730231 PMC7526454

[188] Sonoda N, Imamura T, Yoshizaki T, Babendure JL, Lu JC, Olefsky JM. Beta-Arrestin-1 mediates glucagon-like peptide-1 signaling to insulin secretion in cultured pancreatic beta cells. Proc Natl Acad Sci U S A. 2008;105:6614–6619. doi: 10.1073/pnas.0710402105 18445652 PMC2373360

[189] Campbell JE, Ussher JR, Mulvihill EE, Kolic J, Baggio LL, Cao X, Liu Y, Lamont BJ, Morii T, Streutker CJ, TCF1 links GIPR signaling to the control of beta cell function and survival. Nat Med. 2016;22:84–90. doi: 10.1038/nm.3997 26642437

[190] Zhao P, Liang YL, Belousoff MJ, Deganutti G, Fletcher MM, Willard FS, Bell MG, Christe ME, Sloop KW, Inoue A, Activation of the GLP-1 receptor by a non-peptidic agonist. Nature. 2020;577:432–436. doi: 10.1038/s41586-019-1902-z 31915381

[191] Rasmussen SGF, Choi H-J, Rosenbaum DM, Kobilka TS, Thian FS, Edwards PC, Burghammer M, Ratnala VRP, Sanishvili R, Fischetti RF, Crystal structure of the human β2 adrenergic G-protein-coupled receptor. Nature. 2007;450:383–387. doi: 10.1038/nature06325 17952055

[192] Cherezov V, Rosenbaum DM, Hanson MA, Rasmussen SG, Thian FS, Kobilka TS, Choi HJ, Kuhn P, Weis WI, Kobilka BK, High-resolution crystal structure of an engineered human beta2-adrenergic G protein-coupled receptor. Science. 2007;318:1258–1265. doi: 10.1126/science.1150577 17962520 PMC2583103

[193] Rasmussen SG, DeVree BT, Zou Y, Kruse AC, Chung KY, Kobilka TS, Thian FS, Chae PS, Pardon E, Calinski D, Crystal structure of the beta2 adrenergic receptor-Gs protein complex. Nature. 2011;477:549–555. doi: 10.1038/nature10361 21772288 PMC3184188

[194] Pándy-Szekeres G, Munk C, Tsonkov TM, Mordalski S, Harpsøe K, Hauser AS, Bojarski AJ, Gloriam DE. GPCRdb in 2018: adding GPCR structure models and ligands. Nucleic Acids Research. 2017;46:D440–D446. doi: 10.1093/nar/gkx1109 PMC5753179 29155946

[195] GPCRdb. GPCRdb Structure Statistics. https://gpcrdb.org/structure/statistics. 2024. Accessed 1/13/2024.

[196] Jumper J, Evans R, Pritzel A, Green T, Figurnov M, Ronneberger O, Tunyasuvunakool K, Bates R, Žídek A, Potapenko A, Highly accurate protein structure prediction with AlphaFold. Nature. 2021;596:583–589. doi: 10.1038/s41586-021-03819-2 34265844 PMC8371605

[197] Ghosh E, Kumari P, Jaiman D, Shukla AK. Methodological advances: the unsung heroes of the GPCR structural revolution. Nature reviews Molecular cell biology. 2015;16:69–81. doi: 10.1038/nrm3933 25589408

[198] Shimada I, Ueda T, Kofuku Y, Eddy MT, Wüthrich K. GPCR drug discovery: integrating solution NMR data with crystal and cryo-EM structures. Nat Rev Drug Discov. 2019;18:59–82. doi: 10.1038/nrd.2018.180 30410121 PMC6681916

[199] Elgeti M, Hubbell WL. DEER Analysis of GPCR Conformational Heterogeneity. Biomolecules. 2021;11:778. doi: 10.3390/biom11060778 34067265 PMC8224605

[200] Liu JJ, Horst R, Katritch V, Stevens RC, Wüthrich K. Biased signaling pathways in β2-adrenergic receptor characterized by 19F-NMR. Science. 2012;335:1106–1110. doi: 10.1126/science.1215802 22267580 PMC3292700

[201] Okude J, Ueda T, Kofuku Y, Sato M, Nobuyama N, Kondo K, Shiraishi Y, Mizumura T, Onishi K, Natsume M, Identification of a Conformational Equilibrium That Determines the Efficacy and Functional Selectivity of the μ-Opioid Receptor. Angew Chem Int Ed Engl. 2015;54:15771–15776. doi: 10.1002/anie.201508794 26568421 PMC4722849

[202] Eddy MT, Lee MY, Gao ZG, White KL, Didenko T, Horst R, Audet M, Stanczak P, McClary KM, Han GW, Allosteric coupling of drug binding and intracellular signaling in the A(2A) adenosine receptor. Cell. 2018;172:68–80.e12. doi: 10.1016/j.cell.2017.12.004 29290469 PMC5766378

[203] Shoichet BK, Kobilka BK. Structure-based drug screening for G-protein-coupled receptors. Trends Pharmacol Sci. 2012;33:268–272. doi: 10.1016/j.tips.2012.03.007 22503476 PMC3523194

[204] Yang D, Zhou Q, Labroska V, Qin S, Darbalaei S, Wu Y, Yuliantie E, Xie L, Tao H, Cheng J, G protein-coupled receptors: structure- and function-based drug discovery. Signal Transduct Target Ther. 2021;6:7. doi: 10.1038/s41392-020-00435-w 33414387 PMC7790836

[205] Stein RM, Kang HJ, McCorvy JD, Glatfelter GC, Jones AJ, Che T, Slocum S, Huang XP, Savych O, Moroz YS, Virtual discovery of melatonin receptor ligands to modulate circadian rhythms. Nature. 2020;579:609–614. doi: 10.1038/s41586-020-2027-0 32040955 PMC7134359

[206] Bender BJ, Gahbauer S, Luttens A, Lyu J, Webb CM, Stein RM, Fink EA, Balius TE, Carlsson J, Irwin JJ, A practical guide to large-scale docking. Nat Protoc. 2021;16:4799–4832. doi: 10.1038/s41596-021-00597-z 34561691 PMC8522653

[207] Sadybekov AA, Sadybekov AV, Liu Y, Iliopoulos-Tsoutsouvas C, Huang XP, Pickett J, Houser B, Patel N, Tran NK, Tong F, Synthon-based ligand discovery in virtual libraries of over 11 billion compounds. Nature. 2022;601:452–459. doi: 10.1038/s41586-021-04220-9 34912117 PMC9763054

[208] Slosky LM, Caron MG, Barak LS. Biased allosteric modulators: new frontiers in GPCR drug discovery. Trends Pharmacol Sci. 2021;42:283–299. doi: 10.1016/j.tips.2020.12.005 33581873 PMC9797227

[209] Thal DM, Glukhova A, Sexton PM, Christopoulos A. Structural insights into G-protein-coupled receptor allostery. Nature. 2018;559:45–53. doi: 10.1038/s41586-018-0259-z 29973731

[210] Liu X, Masoudi A, Kahsai AW, Huang LY, Pani B, Staus DP, Shim PJ, Hirata K, Simhal RK, Schwalb AM, Mechanism of β(2)AR regulation by an intracellular positive allosteric modulator. Science. 2019;364:1283–1287. doi: 10.1126/science.aaw8981 31249059 PMC6705129

[211] Liu X, Kaindl J, Korczynska M, Stößel A, Dengler D, Stanek M, Hübner H, Clark MJ, Mahoney J, Matt RA, An allosteric modulator binds to a conformational hub in the β(2) adrenergic receptor. Nat Chem Biol. 2020;16:749–755. doi: 10.1038/s41589-020-0549-2 32483378 PMC7816728

[212] Wisler JW, Rockman HA, Lefkowitz RJ. Biased G protein-coupled receptor signaling: changing the paradigm of drug discovery. Circulation. 2018;137:2315–2317. doi: 10.1161/circulationaha.117.028194 29844068 PMC5984047

[213] Liu X, Ahn S, Kahsai AW, Meng KC, Latorraca NR, Pani B, Venkatakrishnan AJ, Masoudi A, Weis WI, Dror RO, Mechanism of intracellular allosteric β(2)AR antagonist revealed by X-ray crystal structure. Nature. 2017;548:480–484. doi: 10.1038/nature23652 28813418 PMC5818265

[214] Shah SD, Lind C, De Pascali F, Penn RB, MacKerell AD Jr., Deshpande DA. In silico identification of a β(2)-adrenoceptor allosteric site that selectively augments canonical β(2)AR-Gs signaling and function. Proc Natl Acad Sci U S A. 2022;119:e2214024119. doi: 10.1073/pnas.2214024119 36449547 PMC9894167

[215] Ippolito M, De Pascali F, Hopfinger N, Komolov KE, Laurinavichyute D, Reddy PAN, Sakkal LA, Rajkowski KZ, Nayak AP, Lee J, Identification of a β-arrestin-biased negative allosteric modulator for the β(2)-adrenergic receptor. Proc Natl Acad Sci U S A. 2023;120:e2302668120. doi: 10.1073/pnas.2302668120 37490535 PMC10401000

[216] Slosky LM, Bai Y, Toth K, Ray C, Rochelle LK, Badea A, Chandrasekhar R, Pogorelov VM, Abraham DM, Atluri N, β-arrestin-biased allosteric modulator of NTSR1 selectively attenuates addictive behaviors. Cell. 2020;181:1364–1379.e1314. doi: 10.1016/j.cell.2020.04.053 32470395 PMC7466280

[217] Duan J, Liu H, Zhao F, Yuan Q, Ji Y, Cai X, He X, Li X, Li J, Wu K, GPCR activation and GRK2 assembly by a biased intracellular agonist. Nature. 2023;620:676–681. doi: 10.1038/s41586-023-06395-9 37532940

[218] Krumm BE, DiBerto JF, Olsen RHJ, Kang HJ, Slocum ST, Zhang S, Strachan RT, Huang XP, Slosky LM, Pinkerton AB, Neurotensin Receptor Allosterism Revealed in Complex with a Biased Allosteric Modulator. Biochemistry. 2023;62:1233–1248. doi: 10.1021/acs.biochem.3c00029 36917754

[219] Lohse MJ, Bock A, Zaccolo M. G Protein-Coupled Receptor Signaling: New Insights Define Cellular Nanodomains. Annu Rev Pharmacol Toxicol. 2024;64:387–415. doi: 10.1146/annurev-pharmtox-040623-115054 37683278

[220] Buxton IL, Brunton LL. Compartments of cyclic AMP and protein kinase in mammalian cardiomyocytes. J Biol Chem. 1983;258:10233–10239. 6309796

[221] Surdo NC, Berrera M, Koschinski A, Brescia M, Machado MR, Carr C, Wright P, Gorelik J, Morotti S, Grandi E, FRET biosensor uncovers cAMP nano-domains at β-adrenergic targets that dictate precise tuning of cardiac contractility. Nat Commun. 2017;8:15031. doi: 10.1038/ncomms15031 28425435 PMC5411486

[222] Wei W, Smrcka AV. Subcellular β-adrenergic receptor signaling in cardiac physiology and disease. J Cardiovasc Pharmacol. 2022;80:334–341. doi: 10.1097/fjc.0000000000001324 35881897 PMC9452480

[223] Anton SE, Kayser C, Maiellaro I, Nemec K, Möller J, Koschinski A, Zaccolo M, Annibale P, Falcke M, Lohse MJ, Receptor-associated independent cAMP nanodomains mediate spatiotemporal specificity of GPCR signaling. Cell. 2022;185:1130–1142.e1111. doi: 10.1016/j.cell.2022.02.011 35294858

[224] Tsvetanova NG, von Zastrow M. Spatial encoding of cyclic AMP signaling specificity by GPCR endocytosis. Nat Chem Biol. 2014;10:1061–1065. doi: 10.1038/nchembio.1665 25362359 PMC4232470

[225] Irannejad R, Pessino V, Mika D, Huang B, Wedegaertner PB, Conti M, von Zastrow M. Functional selectivity of GPCR-directed drug action through location bias. Nat Chem Biol. 2017;13:799–806. doi: 10.1038/nchembio.2389 28553949 PMC5733145

[226] Kwon Y, Mehta S, Clark M, Walters G, Zhong Y, Lee HN, Sunahara RK, Zhang J. Non-canonical β-adrenergic activation of ERK at endosomes. Nature. 2022;611:173–179. doi: 10.1038/s41586-022-05343-3 36289326 PMC10031817

[227] Daaka Y, Luttrell LM, Ahn S, Della Rocca GJ, Ferguson SS, Caron MG, Lefkowitz RJ. Essential role for G protein-coupled receptor endocytosis in the activation of mitogen-activated protein kinase. J Biol Chem. 1998;273:685–688. doi: 10.1074/jbc.273.2.685 9422717

[228] DeFea KA, Zalevsky J, Thoma MS, Dery O, Mullins RD, Bunnett NW. Beta-arrestin-dependent endocytosis of proteinase-activated receptor 2 is required for intracellular targeting of activated ERK1/2. J Cell Biol. 2000;148:1267–1281. doi: 10.1083/jcb.148.6.1267 10725339 PMC2174299

[229] Mullershausen F, Zecri F, Cetin C, Billich A, Guerini D, Seuwen K. Persistent signaling induced by FTY720-phosphate is mediated by internalized S1P1 receptors. Nat Chem Biol. 2009;5:428–434. doi: 10.1038/nchembio.173 19430484

[230] Calebiro D, Nikolaev VO, Gagliani MC, de Filippis T, Dees C, Tacchetti C, Persani L, Lohse MJ. Persistent cAMP-signals triggered by internalized G-protein-coupled receptors. PLoS Biol. 2009;7:e1000172. doi: 10.1371/journal.pbio.1000172 19688034 PMC2718703

[231] Vilardaga JP, Jean-Alphonse FG, Gardella TJ. Endosomal generation of cAMP in GPCR signaling. Nat Chem Biol. 2014;10:700–706. doi: 10.1038/nchembio.1611 25271346 PMC4417940

[232] Irannejad R, Tomshine JC, Tomshine JR, Chevalier M, Mahoney JP, Steyaert J, Rasmussen SG, Sunahara RK, El-Samad H, Huang B, Conformational biosensors reveal GPCR signalling from endosomes. Nature. 2013;495:534–538. doi: 10.1038/nature12000 23515162 PMC3835555

[233] Bathe-Peters M, Gmach P, Boltz H-H, Einsiedel J, Gotthardt M, Hübner H, Gmeiner P, Lohse MJ, Annibale P. Visualization of β-adrenergic receptor dynamics and differential localization in cardiomyocytes. Proc Natl Acad Sci U S A. 2021;118:e2101119118. doi: doi:10.1073/pnas.2101119118 34088840 PMC8201832

[234] Zaccolo M, Pozzan T. Discrete microdomains with high concentration of cAMP in stimulated rat neonatal cardiac myocytes. Science. 2002;295:1711–1715. doi: doi:10.1126/science.1069982 11872839

[235] Wright CD, Chen Q, Baye NL, Huang Y, Healy CL, Kasinathan S, O’Connell TD. Nuclear alpha1-adrenergic receptors signal activated ERK localization to caveolae in adult cardiac myocytes. Circ Res. 2008;103:992–1000. doi: 10.1161/CIRCRESAHA.108.176024 18802028 PMC2792747

[236] Wright CD, Wu SC, Dahl EF, Sazama AJ, O’Connell TD. Nuclear localization drives α1-adrenergic receptor oligomerization and signaling in cardiac myocytes. Cell Signal. 2012;24:794–802. doi: 10.1016/j.cellsig.2011.11.014 22120526 PMC3393107

[237] Tadevosyan A, Maguy A, Villeneuve LR, Babin J, Bonnefoy A, Allen BG, Nattel S. Nuclear-delimited angiotensin receptor-mediated signaling regulates cardiomyocyte gene expression. J Biol Chem. 2010;285:22338–22349. doi: 10.1074/jbc.M110.121749 20463030 PMC2903375

[238] Boivin B, Chevalier D, Villeneuve LR, Rousseau E, Allen BG. Functional endothelin receptors are present on nuclei in cardiac ventricular myocytes. J Biol Chem. 2003;278:29153–29163. doi: 10.1074/jbc.M301738200 12756260

[239] Lin TY, Mai QN, Zhang H, Wilson E, Chien HC, Yee SW, Giacomini KM, Olgin JE, Irannejad R. Cardiac contraction and relaxation are regulated by distinct subcellular cAMP pools. Nat Chem Biol. 2024;20:62–73. doi: 10.1038/s41589-023-01381-8 37474759 PMC10746541

[240] Liccardo F, Morstein J, Lin TY, Pampel J, Shokat KM, Irannejad R. Selective activation of intracellular beta1AR using a spatially restricted antagonist. bioRxiv. 2023. doi: 10.1101/2023.11.22.568314 PMC11459184 39331410

[241] Abel JJ. On the blood-pressure-raising constituent of the suprarenal capsule. 1897.

[242] Ahlquist RP. A study of the adrenotropic receptors. Am J Physiol. 1948;153:586–600. doi: 10.1152/ajplegacy.1948.153.3.586 18882199

[243] Sutherland EW, Rall TW, Menon T. Adenyl cylase. I. Distribution, preparation, and properties. J Biol Chem. 1962;237:1220–1227. 13918525

[244] Yarden Y, Rodriguez H, Wong SK, Brandt DR, May DC, Burnier J, Harkins RN, Chen EY, Ramachandran J, Ullrich A, The avian beta-adrenergic receptor: primary structure and membrane topology. Proc Natl Acad Sci U S A. 1986;83:6795–6799. doi: 10.1073/pnas.83.18.6795 3018746 PMC386596

[245] Frielle T, Collins S, Daniel KW, Caron MG, Lefkowitz RJ, Kobilka BK. Cloning of the cDNA for the human beta 1-adrenergic receptor. Proc Natl Acad Sci U S A. 1987;84:7920–7924. doi: 10.1073/pnas.84.22.7920 2825170 PMC299447

[246] Palczewski K, Kumasaka T, Hori T, Behnke CA, Motoshima H, Fox BA, Le Trong I, Teller DC, Okada T, Stenkamp RE, Crystal structure of rhodopsin: A G protein-coupled receptor. Science. 2000;289:739–745. doi: 10.1126/science.289.5480.739 10926528

[247] Kang Y, Zhou XE, Gao X, He Y, Liu W, Ishchenko A, Barty A, White TA, Yefanov O, Han GW, Crystal structure of rhodopsin bound to arrestin by femtosecond X-ray laser. Nature. 2015;523:561–567. doi: 10.1038/nature14656 26200343 PMC4521999

[248] Huang W, Masureel M, Qu Q, Janetzko J, Inoue A, Kato HE, Robertson MJ, Nguyen KC, Glenn JS, Skiniotis G, Structure of the neurotensin receptor 1 in complex with β-arrestin 1. Nature. 2020;579:303–308. doi: 10.1038/s41586-020-1953-1 31945771 PMC7100716

[249] Staus DP, Hu H, Robertson MJ, Kleinhenz ALW, Wingler LM, Capel WD, Latorraca NR, Lefkowitz RJ, Skiniotis G. Structure of the M2 muscarinic receptor-β-arrestin complex in a lipid nanodisc. Nature. 2020;579:297–302. doi: 10.1038/s41586-020-1954-0 31945772 PMC7367492

[250] Thomsen ARB, Plouffe B, Cahill TJ 3rd, Shukla AK, Tarrasch JT, Dosey AM, Kahsai AW, Strachan RT, Pani B, Mahoney JP, GPCR-G Protein-β-Arrestin Super-Complex Mediates Sustained G Protein Signaling. Cell. 2016;166:907–919. doi: 10.1016/j.cell.2016.07.004 27499021 PMC5418658

[251] Nguyen AH, Thomsen ARB, Cahill TJ 3rd, Huang R, Huang LY, Marcink T, Clarke OB, Heissel S, Masoudi A, Ben-Hail D, Structure of an endosomal signaling GPCR-G protein-β-arrestin megacomplex. Nat Struct Mol Biol. 2019;26:1123–1131. doi: 10.1038/s41594-019-0330-y 31740855 PMC7108872

[252] Cohn JN, Johnson G, Ziesche S, Cobb F, Francis G, Tristani F, Smith R, Dunkman WB, Loeb H, Wong M, A comparison of enalapril with hydralazine-isosorbide dinitrate in the treatment of chronic congestive heart failure. N Engl J Med. 1991;325:303–310. doi: 10.1056/nejm199108013250502 2057035

[253] Yusuf S, Pitt B, Davis CE, Hood WB, Cohn JN. Effect of enalapril on survival in patients with reduced left ventricular ejection fractions and congestive heart failure. N Engl J Med. 1991;325:293–302. doi: 10.1056/nejm199108013250501 2057034

[254] Yusuf S, Pitt B, Davis CE, Hood WB Jr., Cohn JN. Effect of enalapril on mortality and the development of heart failure in asymptomatic patients with reduced left ventricular ejection fractions. N Engl J Med. 1992;327:685–691. doi: 10.1056/nejm199209033271003 1463530

[255] Cohn JN, Tognoni G. A randomized trial of the angiotensin-receptor blocker valsartan in chronic heart failure. N Engl J Med. 2001;345:1667–1675. doi: 10.1056/NEJMoa010713 11759645

[256] Granger CB, McMurray JJ, Yusuf S, Held P, Michelson EL, Olofsson B, Ostergren J, Pfeffer MA, Swedberg K. Effects of candesartan in patients with chronic heart failure and reduced left-ventricular systolic function intolerant to angiotensin-converting-enzyme inhibitors: the CHARM-Alternative trial. Lancet. 2003;362:772–776. doi: 10.1016/s0140-6736(03)14284-5 13678870

